# Halogens Enhance Haze Pollution in China

**DOI:** 10.1021/acs.est.1c01949

**Published:** 2021-09-30

**Authors:** Qinyi Li, Xiao Fu, Xiang Peng, Weihao Wang, Alba Badia, Rafael P. Fernandez, Carlos A. Cuevas, Yujing Mu, Jianmin Chen, Jose L. Jimenez, Tao Wang, Alfonso Saiz-Lopez

**Affiliations:** † Department of Atmospheric Chemistry and Climate, Institute of Physical Chemistry Rocasolano, CSIC, Madrid 28006, Spain; ‡ Department of Civil and Environmental Engineering, The Hong Kong Polytechnic University, Hong Kong 999077, China; § Institute of Environment and Ecology, Tsinghua Shenzhen International Graduate School, Tsinghua University, Shenzhen 518055, China; ∥ Institute of Environmental Science and Technology (ICTA), Universitat Autònoma de Barcelona (UAB), Barcelona 08193, Spain; ⊥ Institute for Interdisciplinary Science (ICB), National Research Council (CONICET), FCEN-UNCuyo, Mendoza M5502JMA, Argentina; # Research Center for Eco-Environmental Sciences, Chinese Academy of Sciences, Beijing 100085, China; ∇ Department of Environmental Science and Engineering, Fudan University, Institute of Atmospheric Sciences, Shanghai 200433, China; ¶ Cooperative Institute for Research in Environmental Sciences and Department of Chemistry, University of Colorado, Boulder, Colorado 80309, United States

**Keywords:** Reactive halogen species, Secondary aerosol, Haze pollution, Anthropogenic bromine emission, WRF-Chem

## Abstract

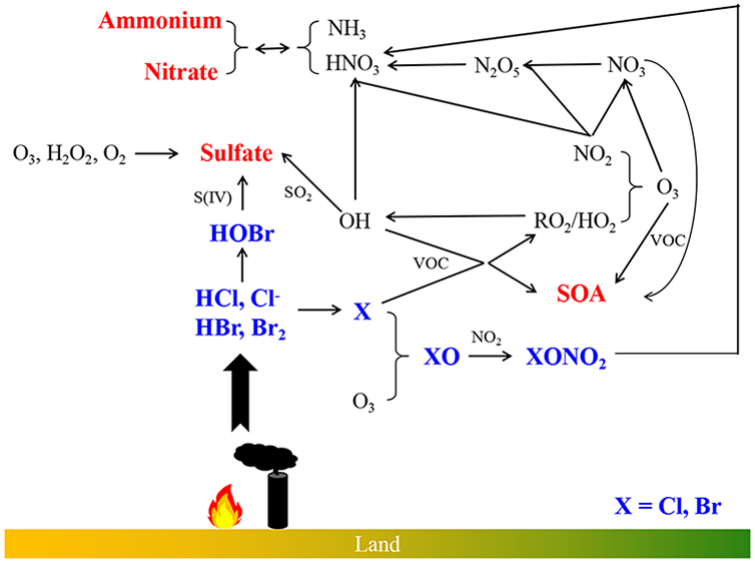

Severe and persistent haze events in northern China, characterized by high loading of fine aerosol especially of secondary origin, negatively impact human health and the welfare of ecosystems. However, current knowledge cannot fully explain the formation of this haze pollution. Despite field observations of elevated levels of reactive halogen species (e.g., BrCl, ClNO_2_, Cl_2_, HBr) at several sites in China, the influence of halogens (particularly bromine) on haze pollution is largely unknown. Here, for the first time, we compile an emission inventory of anthropogenic bromine and quantify the collective impact of halogens on haze pollution in northern China. We utilize a regional model (WRF-Chem), revised to incorporate updated halogen chemistry and anthropogenic chlorine and bromine emissions and validated by measurements of atmospheric pollutants and halogens, to show that halogens enhance the loading of fine aerosol in northern China (on average by 21%) and especially its secondary components (∼130% for secondary organic aerosol and ∼20% for sulfate, nitrate, and ammonium aerosols). Such a significant increase is attributed to the enhancement of atmospheric oxidants (OH, HO_2_, O_3_, NO_3_, Cl, and Br) by halogen chemistry, with a significant contribution from previously unconsidered bromine. These results show that higher recognition of the impact of anthropogenic halogens shall be given in haze pollution research and air quality regulation.

## Abstract 2

Anthropogenic emission of reactive halogens boosts the production of secondary aerosols leading to air quality degradation and calling for regulation.

## 1 Introduction

Haze pollution has been threatening the health of millions of people in China for the past two decades. Haze pollution is characterized by an extremely high loading of fine aerosol (also known as PM_2.5_, particulate matter with aerodynamic diameter ≤2.5 μm), and especially that of secondary aerosol (formed from gases in the atmosphere as opposed to the primary aerosols that are particles directly emitted from sources).^1^ Secondary aerosols are mainly secondary organic aerosol (SOA), sulfate, nitrate, and ammonium. SOA comprises many oxidation products of various volatile organic compounds (VOCs) by oxidants, that is, OH, O_3_, and NO_3_.^2^ Sulfate is formed from the gaseous oxidation of SO_2_ by OH, aqueous reactions by H_2_O_2_, O_3_, and O_2_ catalyzed by transition metal ions (TMIs) in cloud droplets, and heterogeneous reactions on aerosol.^3−5^ Nitrate is produced from NO_*x*_ chemical processes involving OH and O_3_.^6^ Ammonium aerosol (in the form of ammonium sulfate and ammonium nitrate) is determined by the availability of sulfuric acid, nitric acid (HNO_3_), ammonia (NH_3_), and aerosol pH. Details of traditional formation channels of secondary aerosols are summarized in Supporting Information (SI) Text S1. The current aerosol mechanisms are not able to fully explain the observed secondary aerosols in haze pollution in China calling for a better understanding of their formation processes.^7^

In addition to the well-known role of ocean-emitted halogens in photochemistry in the polar and marine troposphere,^8,9^ anthropogenic halogens are increasingly recognized to play a role in continental air pollution,^10−17^ by perturbing the levels of oxidants and atmospheric pollutants, including HO_*x*_ (OH and HO_2_), NO_*x*_ (NO and NO_2_), VOCs, O_3_, sulfur species, etc. Briefly, halogens enhance the production of oxidants (OH, O_3_, and NO_3_) by igniting photochemistry (R1–R7). Halogens also directly destroy O_3_ via reactions of halogen atoms with O_3_ (R8) and indirectly destroy O_3_ via reducing the level of NO_2_ (R9–R10, R5). The presence of abundant VOCs and NO_*x*_ species in the polluted region facilitates the reactions R1–R7 leading to an enhancement effect of halogens on oxidants; meanwhile, in the clean environment, reactions R8–R9 directly destroy O_3_ (the main source of OH radicals) and dominate the effect of halogens on oxidants, decreasing oxidation capacity.
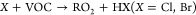
R1
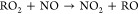
R2
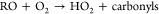
R3
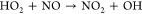
R4
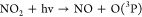
R5
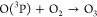
R6
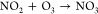
R7
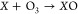
R8
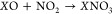
R9
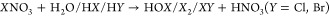
R10

In the past few years, observational studies have reported elevated levels of halogen species in China. The majority of these reports focused on ClNO_2_^18−22^ and a few on Cl_2_^23,24^ and bromine species (BrCl, HOBr, Br_2_, and HBr).^25,26^ A few modeling studies evaluated the impacts of chlorine chemistry on O_3_ and PM_2.5_.^11,14^ However, the overall impact of anthropogenic halogens (particularly bromine) on aerosols and haze pollution in China remains poorly understood.

In this study, for the first time, we quantify the collective role of halogens in haze pollution with a regional model, Weather Research and Forecasting model coupled with Chemistry (WRF-Chem), updated to include comprehensive reactive halogen chemistry and natural halogen sources,^27^ as well as anthropogenic chlorine^28^ and bromine emissions. The bromine emission presented in this work is the first compilation of anthropogenic inorganic bromine sources in the world. The bromine emission compilation method, the WRF-Chem model, and WRF-Chem simulations are summarized in Section 2. The simulation results of the halogen impacts on secondary aerosols are discussed in Section 3.

## 2 Material and Method

### 2.1 Anthropogenic Bromine Emission Inventory

A bottom-up emission inventory of reactive bromine species from coal combustion activities, including power plants, industrial processes (e.g., cement, iron and steel, brick, lime production), industrial boiler, and residential burning, in China in the year 2017 is proposed in this study. An emission factor method was applied to estimate total bromine emissions, based on the following equations:
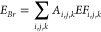
1
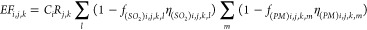
2where *A* is coal consumption, which was obtained from Chinese official energy statistics^29^ or calculated as the products of industrial outputs and the corresponding coal intensities, as described in Fu et al.^28^ Residential coal consumptions have been revised based on previous surveys,^30^ due to high uncertainties in statistic data for this sector. EF is the emission factor. *C* is Br content in coal. Peng et al.^31^ reported values ranging from 0.12 to 69.66 μg/g in 305 coal samples from 27 provinces in China. The highest value, 69.66 μg/g, was used for raw coal in this study, to account for other potential unknown Br sources which are not included in this study. Even though the current estimation represent an upper limit for Br emission from coal combustion, the total bromine emission over China is not overestimated, as indicated by the validation results of reactive bromine species in our study (Section 2.3). Br contents in cleaned coal and briquette coal were calculated as described in Fu et al.^28^ The Br release rates (*R*) for different combustion technology and processes were derived from previous measurements and studies, as listed in SI Table S1. *f*_(SO2)_ and *f*_(PM)_ are application rates of conventional SO_2_ and PM emission control technologies, obtained from the emission database established in previous studies.^32,33^ The Br removal efficiencies (η_(SO2)_ and η_(PM)_) were set as 13.4% for electrostatic precipitator and fabric filter, 50% for wet scrubber, and 92.5% for flue gas desulfurization, based on the previous measurements for different control devices.^34−40^*i*, *j*, *k*, *l*, *m* represent the province, sector, technology type, SO_2_ emission control technology and PM emission control technology, respectively. HBr and Br_2_ were reported to be the two dominant emitted Br species,^37,41^ whose proportions were set as 70% and 30%, respectively, in this study. The annual emissions were distributed into each month based on the factors in Wang et al.^42^ for residential burning and equally for other sectors.

**Figure 1:**
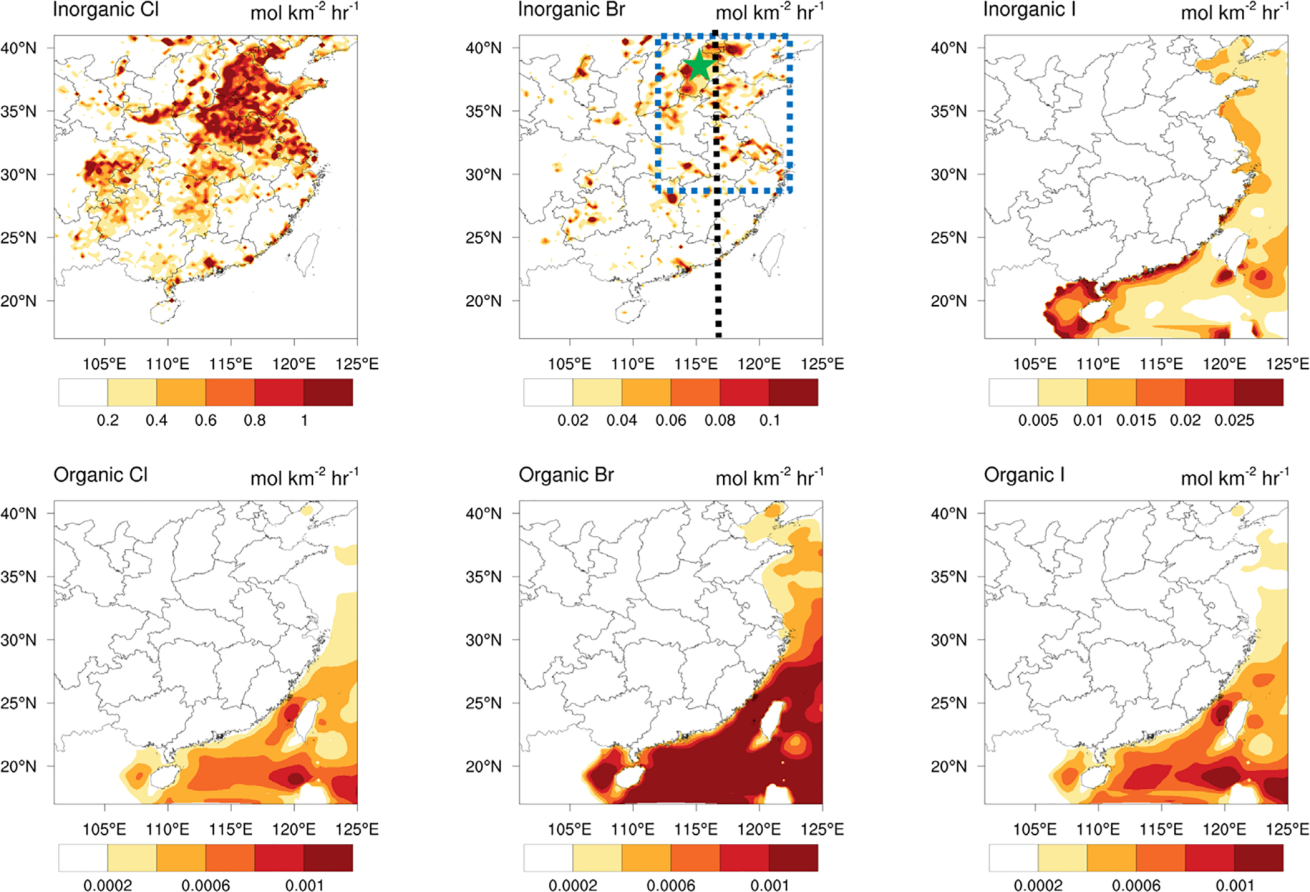
Average emissions of Cl, Br, and I species (mol km^–2^ hr^–1^). The blue rectangle represents the region of interest, northern China. The green star stands for the location of the Wangdu campaign (used in model evaluation in SI Text S4). The black line (longitude = 116.3°) is the location of the vertical cross-section shown in Figure 4.

The overall emission intensity of halogen species during the simulation period is shown in Figure 1, in which anthropogenic inorganic chlorine (>0.2 mol km^–2^ hr^–1^ in polluted areas) and bromine species (>0.02 mol km^–2^ hr^–1^) dominate the halogen emission over China. The bromine emission inventory compiled in the current work is the first anthropogenic inorganic bromine source and there is no relevant emission data set to compare. We evaluate this newly proposed emission data set by comparing the WRF-Chem simulated bromine species, BrCl, HOBr, and Br_2_, with the respective observed values, as shown in SI Text S4.

### 2.2 WRF-Chem Model

WRF-Chem is a regional air quality model commonly used to investigate air pollution and its formation mechanism.^43^ The SI spreadsheet file shows the chemical mechanism (photolysis, gas-phase reactions, and multiphase reactions) used in the present study.^27,44−48^ As to the aerosol phase mechanism, there are three main processes, including nucleation, coagulation, and gas-particle partitioning. The details of the chemical processes in the standard WRF-Chem model that are relevant to the secondary aerosol formation can be found in SI Text S1. Here we describe the new reactions and chemical processes that we added in WRF-Chem in the present study.

a. SOA. We followed the volatility basis set (VBS) framework^49−51^ and utilized the Cl-initiated SOA yield data reported in previous chamber studies^52−57^ to propose a new set of Cl-initiated SOA yield parametrizations for their use in chemical transport models (SI Text S2; Figure S1; Table S2). Two Br-initiated VOCs oxidations (R17–R18) are also considered to result in SOA formation using the same yield of Cl-initiated reactions for the lack of Br-initiated SOA yield data. In the following reactions, BIGALK, ISOP, APIN, BPIN, and LIMON represent alkanes with more than 3 carbon atoms, isoprene, α-pinene, β-pinene, and limonene, respectively.

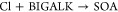
R11
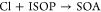
R12
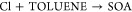
R13
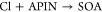
R14
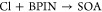
R15
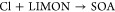
R16
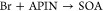
R17
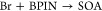
R18

b. Sulfate. The formation of sulfate aerosol through the uptake of HOBr on the aqueous phase S(IV)-containing aerosol.^58−61^ We followed Chen et al.^60^ and the references therein to adopt a rate of 5.0 × 10^9^ M^–1^ s^–1^ for HOBr+SO_3_^2–^ reaction, and we used the recently proposed rate of 2.6 × 10^7^ M^–1^ s^–1^ by Liu and Abbatt^61^ for HOBr+HSO_3_^–^ reaction.

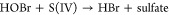
R19

c. We added a few heterogeneous reactions of chlorine and bromine species on the aerosol surface. According to Abbatt et al.,^62^ the solubility and reactivity of BrNO_2_ are substantially higher than that of ClNO_2_, which potentially enables BrNO_2_ to be taken up on the aerosol surface. In a polluted environment during winter as in northern China (∼10 ppbv O_3_ and ∼30 ppbv NO_2_), BrNO_2_ is the dominant form of gaseous bromine if its uptake reaction is not activated. Two multiphase channels of BrCl formation from HOCl and BrNO_3_ are also added.

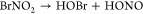
R20
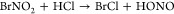
R21
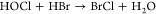
R22
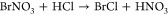
R23

d. We also introduced a photoenhanced uptake coefficient for the heterogeneous bromine reactions to mimic the good correlation between the photolysis rate and the level of bromine species (see SI Text S4).

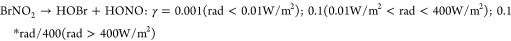

e. Several Br atom initiated VOC oxidation channels. MVK and MACR represent methyl vinyl ketone and methacrolein, respectively. Note that the reactions of Br atom with ISOP and LIMON are not included in the current study and should be considered in future works which will further enhance the halogen effects on VOC oxidation and SOA formation.

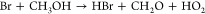
R24
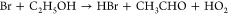
R25
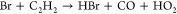
R26
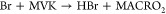
R27
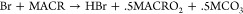
R28
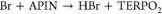
R29
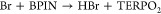
R30Here we acknowledge that a few previously reported potential pathways of secondary aerosols are not included in our chemical scheme. For instance, Cheng et al.^4^ proposed an aqueous pathway of sulfate formation from NO_2_ oxidation but we expect such a pathway would not have a large effect in northern China during our simulated period due to the low RH (SI Figure S2). Recent works^5,63^ also suggested that the pH level in northern China might be too low for the NO_2_ oxidation pathway to occur. The research on SOA formation from various chemical pathways (e.g., oxidation of primary organic aerosol) has received attention in the past few years^64,65^ but these new channels are not considered in the present work. The omission of these very recent developments of SOA could be the cause of the underestimated SOA in our study (see Section 2.3). However, we note that current chemical transport models are subject to large uncertainty in secondary aerosol simulation particularly under polluted conditions. The purposes of this study are (1) to implement the most up-to-date halogen sources and chemistry, (2) to quantify the relative impact of halogens in the formation of fine aerosol, and (3) to motivate further research oriented to reproduce the observed high levels of secondary aerosols during haze pollution in China.

### 2.3 WRF-Chem Simulations

The domain of the WRF-Chem simulation covers the major city clusters in China with a grid size of 27 km. The simulation period is the same as the Wangdu campaign, during which comprehensive reactive halogen species were measured,^25^ i.e. December 9 to 31, 2017, with an extra spin-up period of 20 days (November 20 to December 8, 2017). The physical and chemical parametrizations adopted in this study are listed in SI Table S3.

Several data sets were used to initiate and drive the WRF-Chem model. Data set ds083.2 provided by Nation Centres for Environmental Prediction (NCEP) was used as the meteorological input. Data sets ds351.0 and ds461.0 were used for the data assimilation of meteorological simulation in the WRF model. The output from the CAM-Chem model, a global chemistry and climate model, was used as chemical initial and boundary conditions following Li et al.^15^ As to the anthropogenic emissions, a widely used emission inventory, MEIC (www.meicmodel.org), is adopted for the routine air pollutants (NO_*x*_, SO_2_, CO, VOC, NH_3_, PM_2.5_, PM_10_). We also applied an open fire emission inventory, the Fire Inventory from NCAR (FINN).^66^ Natural halogen emissions were estimated online using the mechanism described in Badia et al.^27^ Anthropogenic chlorine emissions from the burning activities of coal, biomass, and municipal solid waste compiled by Fu et al.^28^ are adopted. The anthropogenic bromine emission inventory developed for this work (Section 2.1) is also employed.

We conducted two major simulations, BASE and HAL, whose results are discussed in detail. BASE case includes no halogen sources nor chemistry and only the reactions in the standard WRF-Chem configuration.^44−47^ The HAL case includes a full set of comprehensive halogen sources and chemistry (Archer-Nicholls;^48^ Badia et al.;^27^ this work) described in Section 2.2 and SI Text S1. The difference in atmospheric compositions between BASE and HAL is the impact of the overall halogen sources and chemistry. We also ran five additional sensitivity cases, namely, no_Cl_emi, no_Br_emi, no_BrNO_2__uptake, fixed_gamma, no_ClBr_SOA, to identify the key factors determining the overall halogen effect on secondary aerosol production. The difference between these scenarios is shown in SI Table S4 and Text S3.

We use the observational data reported in Peng et al.^25^ to evaluate the model performance. The average observed bromine species (BrCl+HOBr+2 × Br_2_) during the campaign is 105 pptv; our WRF-Chem model generally simulated the magnitude and temporal variation with an average of 71 pptv (SI Figure S8; Text S4). The simulated ClNO_2_ reproduces well the level and temporal trend of the observation, although with a mean underestimation of 25%. Our modeling results present some discrepancies on the hourly concentration profile due to the omission of local sources, which are a common issue of regional modeling studies. However, the general underestimation in chlorine and bromine abundances in WRF-Chem suggests that the halogen effects reported in this study should be considered as a lower limit in this region. Note that our model setup provides a good representation of the gas phase chemistry (SI Figure S9) because the simulated gaseous pollutants (NO_2_, SO_2_, and O_3_), total reactive nitrogen (NO_*y*_), and oxidant precursors (HONO and H_2_O_2_) are very close to the observations, although the simulated NH_3_ is moderately lower than the measurement. As to the fine aerosol and its composition (SI Figure S10), the simulated PM_2.5_, sulfate, and black carbon are in line with the observation, and the modeled SOA is underestimated, and the simulated nitrate and ammonium aerosols are significantly overestimated. The underestimation of SOA could be due to the omission of local sources and other chemical formation channels which are not included in this study. The good simulation of NO_2_ and NO_*y*_, the underestimation of NH_3_, and the overestimation of nitrate and ammonium suggest that the gas-particle partitioning between ammonium nitrate aerosol and HNO_3_ and NH_3_ might be the cause for the overestimated nitrate and ammonium aerosol. We explore this issue in more detail in SI Text S4.

Overall, this updated WRF-Chem model reasonably simulates the level and temporal variation of the reactive halogen species, oxidants, and air pollutants. The overestimation of nitrate and ammonium and underestimation of SOA is not related to halogens and will not affect the aim of the present work, that is, the halogen impacts on haze pollution. Therefore, the current setup is suitable to quantify the relative impact of reactive halogen chemistry on secondary aerosol production and haze pollution in China.

## 3 Results and Discussion

### 3.1 Simulated Halogens in China

**Figure 2:**
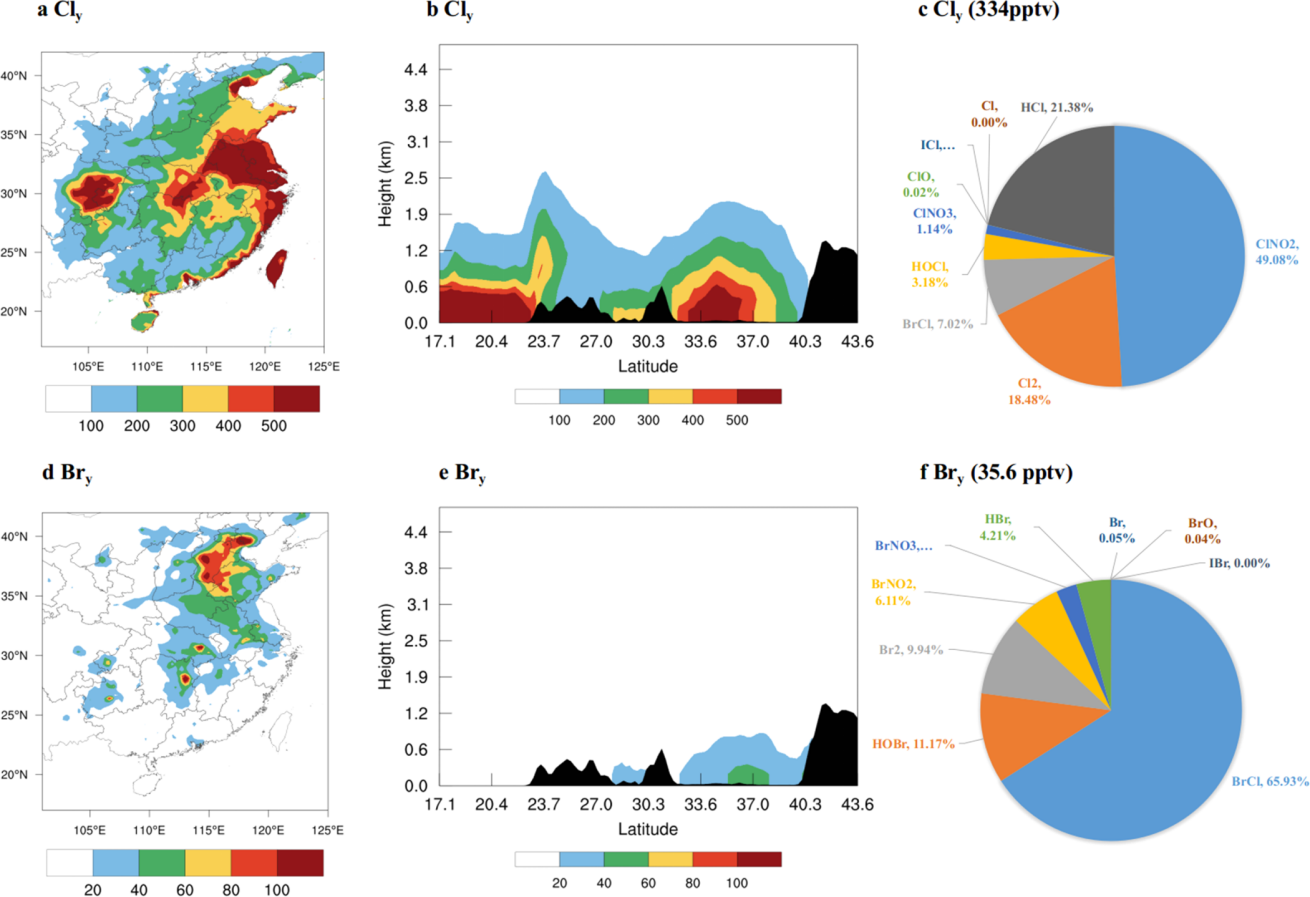
Average simulated Cl_*y*_ (pptv; a, surface; b, cross-section) and Br_*y*_ (pptv; d; e) in the HAL scenario. Partitioning of Cl_*y*_ (c) and Br_*y*_ (f) in the HAL scenario in northern China and the mixing ratios of Cl_*y*_ and Br_*y*_ shown in the brackets are the average values in northern China (location shown in Figure 1). The black shaded area in insets b and e is the mountainous region.

The simulated spatial (horizontal and vertical) distribution and partitioning of halogens in the HAL scenario are depicted in Figure 2. The modeled Cl_*y*_ (total gaseous inorganic chlorine = BrCl+ClNO_2_+2*Cl_2_+HOCl+ClNO_3_+ICl+ClO+Cl+HCl) is elevated (>300 pptv) in northern China, along the coast, and in southwestern China; whereas the estimated Br_*y*_ (total gaseous inorganic bromine = BrCl+BrNO_2_+2*Br_2_+HOBr+BrNO_3_+IBr+BrO+Br+HBr) shows large mixing ratios (>60 pptv) in northern and central China and much lower in other regions. The average partitioning of Cl_*y*_ in northern China shows that ClNO_2_ is the dominant species (∼50%), whereas HCl, Cl_2_, BrCl, and HOCl contribute about 50%. A large scale observation campaign of reactive halogen species was conducted at Wangdu site (location shown in Figure 1) in northern China,^25^ reporting the first set of inorganic bromine species in China. During the Wangdu campaign, BrCl is the predominant chlorine species,^25^ and our simulation (in the same period) also predicted higher BrCl levels than other chlorine species on most days (SI Text S4). As to Br_*y*_, BrCl is also the most abundant species which contributes ∼60%, whereas HOBr and Br_2_ take up ∼20%. Our simulated halogen species are in line with the available observational reports of reactive bromine species in China by Peng et al.^25^ and Fan et al.^26^ and also within the ranges in previous studies of chlorine species in China.^19,23,24^

### 3.2 Halogen Impact on Oxidants

The spatial distribution of oxidants and their changes due to halogens are shown in SI Figure S3. The addition of halogens induces large increases of OH (+54% or +0.01 pptv) and HO_2_ (+160% or +0.56 pptv) in northern China (R1–R4). Similar increases (+26% to +73% in OH, HO_2_, and RO_2_ radicals) due to halogens were reported at Wangdu site in northern China during the same period using a box model constrained with the observed halogen species.^25^ Note that the enhancement of HO_*x*_ by halogens helps to close the gap between observed and the under-predicted HO_*x*_ radicals by many models.^67^ O_3_ is enhanced by halogens in northern China (+27% or +5.4 ppbv) suggesting that the O_3_ enhancing pathways (R1–R6) exceed those that destroy O_3_ (R8-R10, R5). The large increase of O_3_ due to the reactive halogen chemistry presented here, which is not fully considered in models, could partially explain the “fast photochemistry” observed in winter in northern China.^68^ NO_3_ is also significantly increased due to halogens by 23% or 0.41 pptv in northern China (R7). The enhancement in these oxidants is mostly caused by the Cl and Br-initiated VOCs oxidation (R1) leading to additional production of OH and HO_2_ (R2–R4) and subsequently O_3_ (R5–R6) and NO_3_ (R7). The presence of halogen species transforms a fraction of NO_*x*_ into halogen nitrates (R9) which also contributes to the increase in O_3_ in this polluted region due to the nonlinear O_3_ chemistry. A previous modeling study^15^ provided a more detailed analysis of the halogen impacts on oxidants in China.

### 3.3 Halogen Impact on Secondary Aerosol

**Figure 3:**
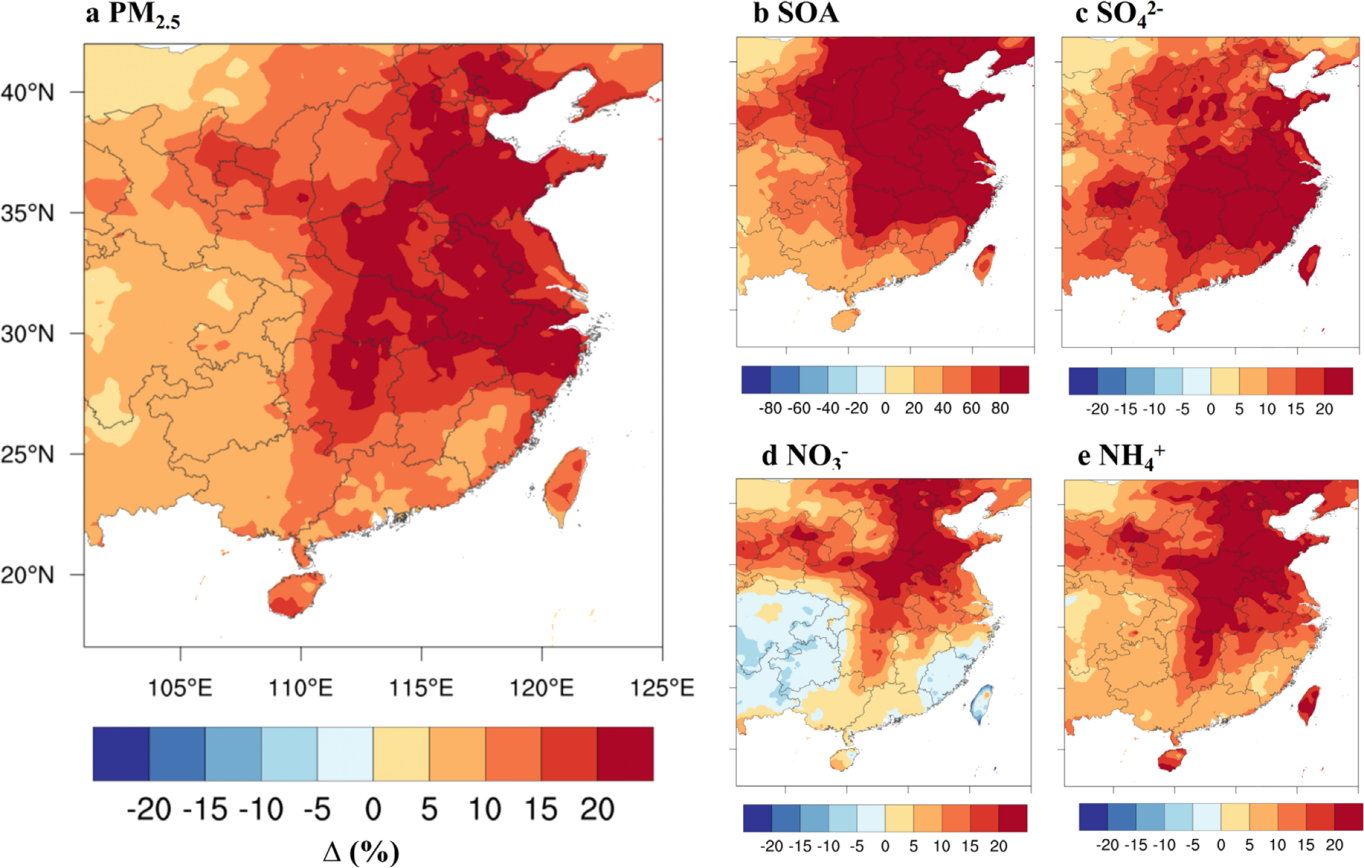
Average simulated impact (in percentage) of reactive halogen chemistry on (a) fine aerosol and its major components (b SOA; c sulfate; d nitrate; e ammonium) at the surface in China from December 9 to December 31, 2017. Note the difference in the color scales of SOA changes. Results for the BASE and HAL simulations and their difference (in μg m^–3^) are shown in SI Figure S4. The change (%) in the total fine aerosol is smaller than those (%) in the secondary aerosol because some parts of the fine aerosol are primary aerosol which remains constant in the BASE and HAL cases.

Figure 3 shows the simulated halogen impacts (in percentage) on fine aerosol and its secondary components (SOA, sulfate, nitrate, and ammonium) over China. The simulated levels of aerosols in BASE and HAL cases and their difference (in μg m^–3^) are shown in SI Figure S4. The average increase due to halogen chemistry in northern China (the area shown as the blue rectangle in Figure 1) is 21% (21.7 μg m^–3^) for the total fine aerosol, including 136% (8.7 μg m^–3^) for SOA, 21% (2.2 μg m^–3^) for sulfate, 19% (6.4 μg m^–3^) for nitrate, and 23% (3.2 μg m^–3^) for ammonium. Of particular interest is the modeled SOA. Although our simulated SOA in the HAL case is still lower than the observation (see SI Text S4), the addition of halogens brings the simulated SOA concentration closer to the observations. Indeed, the halogen-mediated SOA formation helps with the current model under predictions of urban SOA.^64^

**Figure 4:**
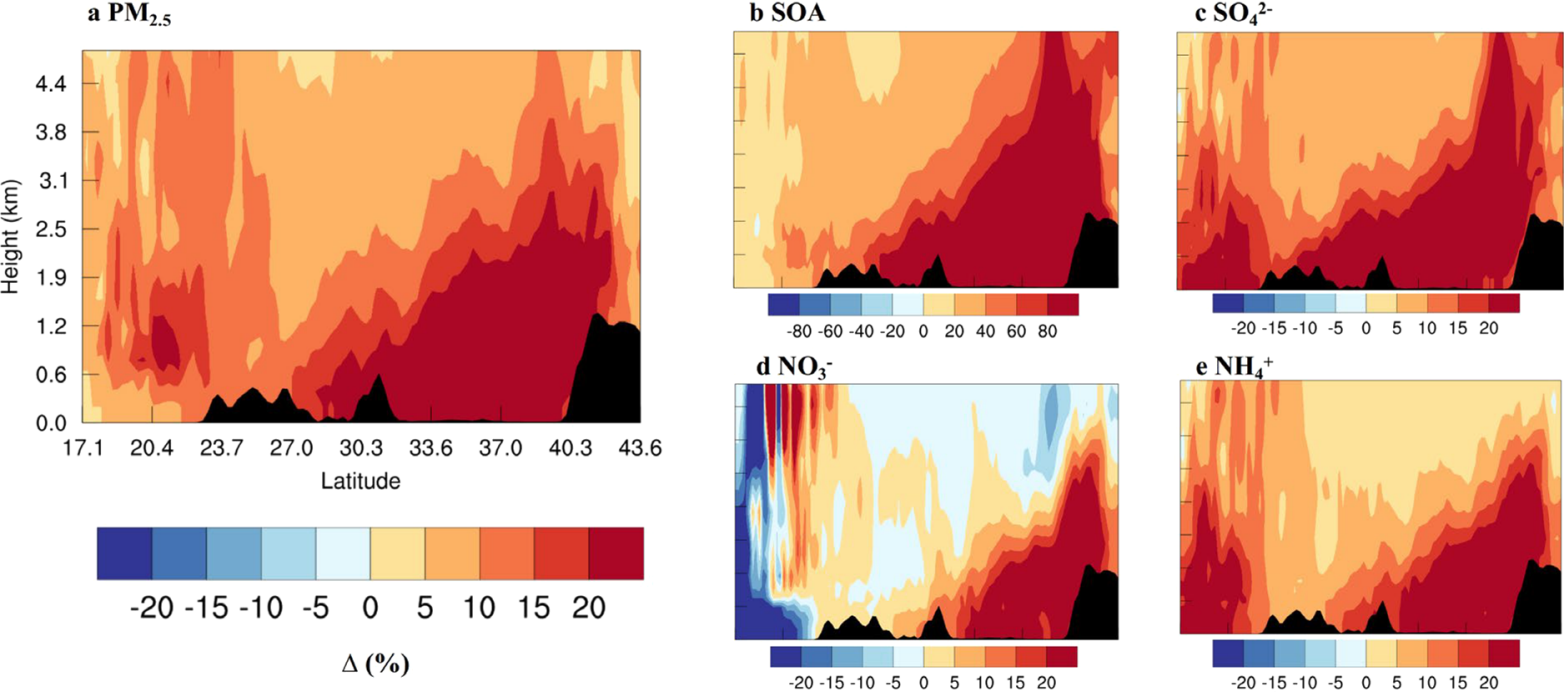
Average simulated impact (%) of reactive halogen chemistry on (a) fine aerosol and its major components (b SOA; c sulfate; d nitrate; e ammonium) in the south-north vertical cross-section (see location in Figure 1). The black shaded area is the mountainous region. Note the difference in the color scale of SOA changes. Results for the BASE and HAL simulations and their difference (in μg m^–3^) are shown in SI Figure S5.

We find that halogens influence the formation of fine aerosols throughout the planetary boundary layer (PBL) and extending well into the free troposphere (see Figure 4 for percentage changes and SI Figure S5 for absolute changes and the levels of aerosols in the BASE and HAL cases). Thereby, the halogen effect on secondary aerosol formation is not restricted to the emission region and boundary layer because chemical recycling processes (both gaseous and multiphase) occurring during the transport extend the area and altitude of influence. While halogen chemistry induces the largest absolute changes near the surface in northern China (SI Figure S5), the higher percentage difference is observed in the upper parts of the PBL (Figure 4). This is because northern China is “flooded” of air pollutants (e.g., NO_*x*_) which suppress the surface levels of O_3_, NO_3_, N_2_O_5_, and hence *X*(=Cl, Br) and *X*O radicals; whereas in the upper PBL and the free troposphere, the lower NO_*x*_ and increased O_3_ allows for the more efficient recycling of gas-phase halogens, thereby further enhancing secondary aerosol production. In the cleaner environment (open ocean and free troposphere), halogens reduce O_3_ and OH, therefore, decreasing nitrate aerosol production; halogens also redirect some of the N_2_O_5_ heterogeneous reactions to form ClNO_2_/BrNO_2_ (N_2_O_5_+Cl^–^(aq) → NO_3_^–^(aq)+ClNO_2_; RS11 in the SI Text S1) instead of HNO_3_ when no halogens are present (N_2_O_5_+H_2_O(aq) → 2HNO_3_(aq); RS7), and hence reduce the production of nitrate aerosol within the HAL case. Such a decrease of nitrate aerosol due to chlorine reactions with N_2_O_5_ has been reported recently.^69^

Halogens also modify the diurnal variation of the aerosols and their fractions in PM_2.5_ at the surface in northern China (SI Figure S6). In particular, the fraction of SOA in the fine aerosol increases from 7.6% in the BASE case to 14.3% in the HAL case. The relative contributions of primary aerosols to the total fine aerosol, on the other hand, are decreased (from 14.7% to 11.7% for primary OA and 7.3% to 5.8% for BC). The addition of halogen chemistry also substantially increases the fraction of chloride in the fine aerosol (from 0.1% to 1.1%). The increased chloride has recently been reported to sustain aerosol growth by enhancing ammonium aerosol and water uptake in a polluted area in India.^16^

### 3.4 Chemical Pathways for Halogen Impact on Secondary Aerosol Formation

**Figure 5:**
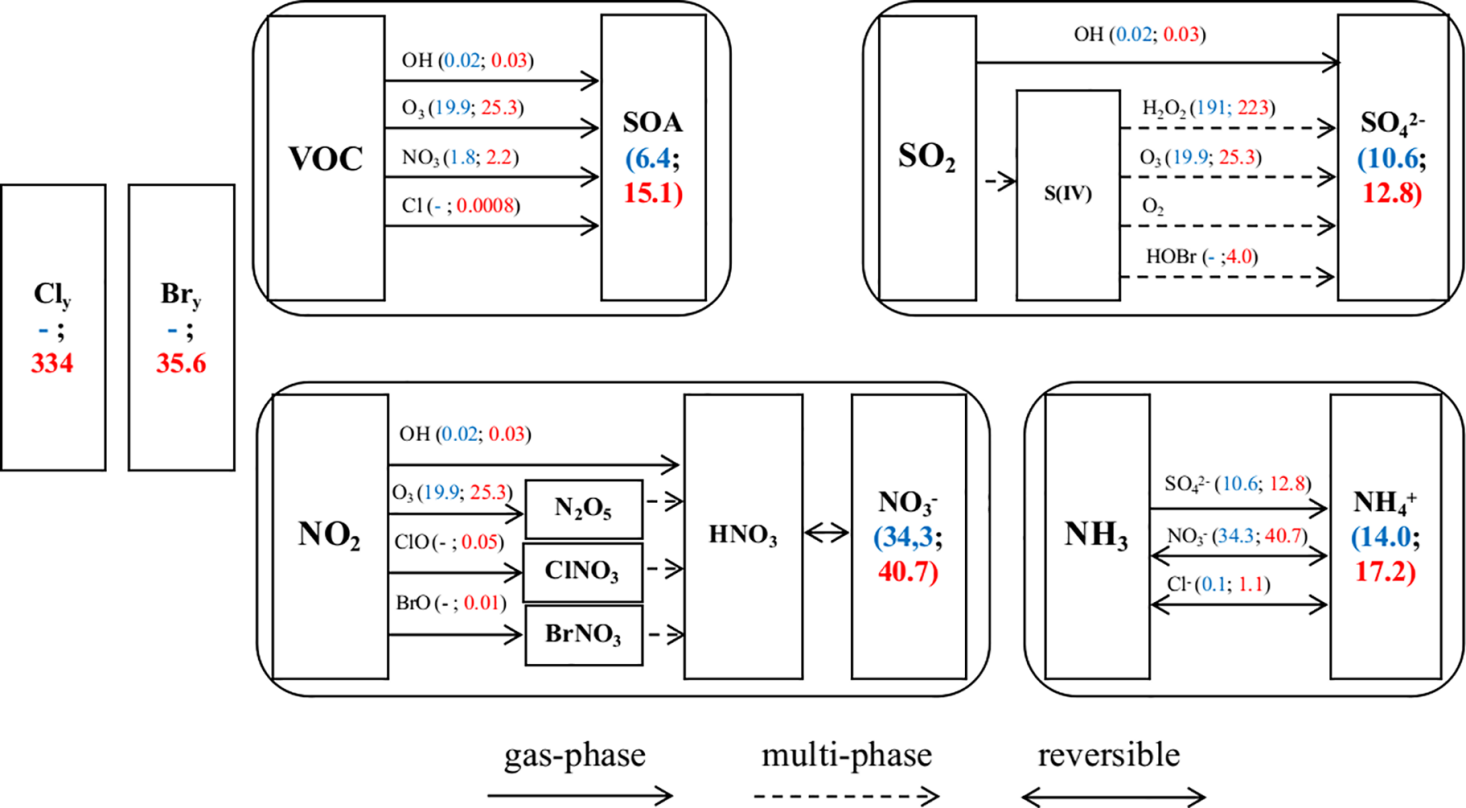
Formation pathways of secondary aerosols (SOA, sulfate, nitrate, and ammonium). The mixing ratios of gaseous species are the average values in northern China (location shown in Figure 1) in pptv except for O_3_ which is in ppbv; the concentrations of particulate species are the average values in northern China in μg m^–3^. Different colors represent results from the BASE (in blue) and HAL (in red) cases, e.g, OH (0.02; 0.03) represents that the OH mixing ratio in the BASE case is 0.02 pptv and that in HAL case is 0.03 pptv. Note that the level of O_2_ is not affected by halogens. Note that the HOBr+S(IV) reaction takes place in aerosol water.

Halogens impact the concentrations of the conventional oxidants (OH, O_3_, and NO_3_) and thus the rates of the reactions of these oxidants with gas-phase pollutants (e.g., VOC, SO_2_, NO_*x*_) ultimately altering the formation of the secondary aerosols. In addition to this dominant indirect effect, halogens also participate in the direct production processes of all secondary aerosols. Figure 5 summarizes the main chemical channels to form secondary aerosol together with the average levels of oxidants and secondary aerosols in the BASE and HAL scenarios in northern China.

Halogens enhance the production of SOA by increasing the mixing ratios of OH, O_3_, and NO_3_, and adding two new radicals (Cl and Br) (Figure 5). Sulfate aerosol is increased through enhancing OH, H_2_O_2_ (product of HO_*x*_ self-productions), and O_3_, and the introduction of a new sulfate formation channel initiated by HOBr (Figure 5). The nitrate aerosol concentrations are enhanced both directly through halogen oxides (R9–R10) and indirectly via their halogen-mediated enhancement of the levels of OH and O_3_ (R1–R6). The increases of sulfate and nitrate and the addition of chloride, with the presence of NH_3_, induce a noticeable increase of ammonium via gas-particle partitioning (e.g., NH_3_ + HNO_3_ → NH_4_NO_3_ aerosol). Liu et al.^70^ suggested that PM_2.5_ in China could be reduced by 11%–17% provided that a 50% reduction of NH_3_ and a 15% reduction of SO_2_ and NO_*x*_ are achieved. Here we note that by omitting reactive halogens, one might leave out an equivalent fraction of the aerosols formation rates in air quality model forecasting (∼20% of ammonium and ∼20% of PM_2.5_).

**Figure 6:**
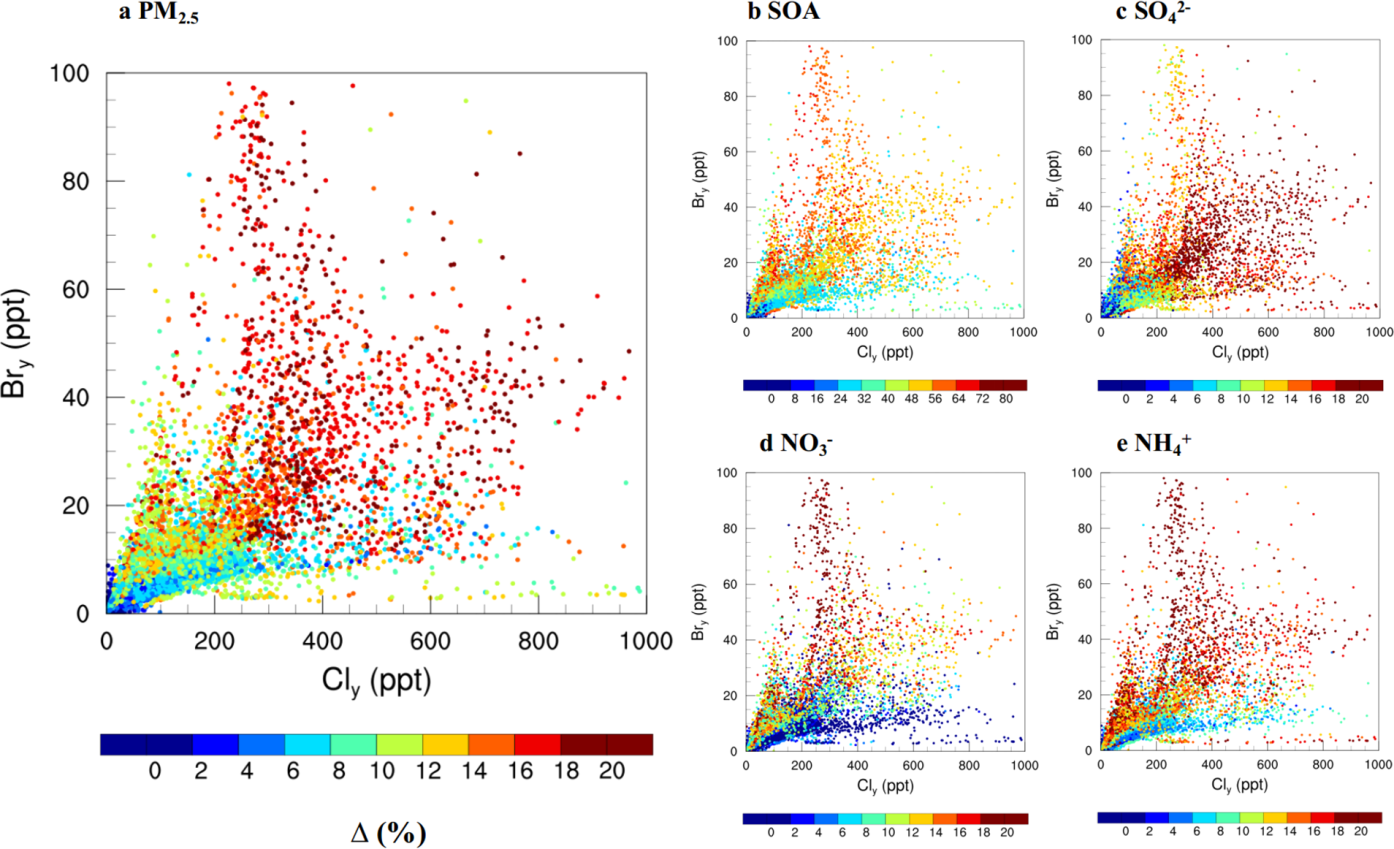
Relationship between the aerosols changes (a, PM_2.5_; b, SOA; c, sulfate; d, nitrate; and e, ammonium) and the level of chlorine and bromine species at the surface in China. Note that the negative changes in nitrate in southeast and southwest China is omitted in this figure. The scale of SOA is different from other panels.

Figure 6 further shows the relationship of aerosol changes (in all locations within the simulation domain) to the corresponding levels of Cl_*y*_ and Br_*y*_. Our results show that larger Cl_*y*_ levels at a given Br_*y*_ level lead to larger changes of aerosols although this sensitivity is reduced for Cl_*y*_ levels over 300 pptv; larger Br_*y*_ mixing ratios at a given Cl_*y*_ level result in larger enhancements on secondary aerosol, though the changes in the aerosol are insensitive to Br_*y*_ larger than 50 pptv. An exception is sulfate, whose largest enhancement does not correlate with highest Br_*y*_ because the multiphase reaction of HOBr+S(IV) → HBr+Sulfate requires humid conditions (Section 2.3; R19) but a large fraction of northern China (the region with the highest bromine) has a low RH during the simulation period (SI Figure S2). An empirical connection of Cl_*y*_ to fine aerosol changes could be drawn from our simulation, that is, > 300 pptv Cl_*y*_ leads to ∼20% enhancement in PM_2.5_ and similarly, > 50 pptv Br_*y*_ leads to ∼20% enhancement in fine aerosol implying that per unit of mixing ratio, bromine leads to a larger impact on aerosols compared to that of chlorine. Such phenomena will be discussed in more detail in the next section using sensitivity simulations.

### 3.5 Sensitivity of Halogen-Driven Aerosol Changes to Key Factors

In light of the complexity and the uncertainty of the halogen effects on aerosol concentration and composition, we conducted five additional sensitivity simulations (SI Table S4) to quantify the sensitivity of halogen impact on aerosol to the key parameters and processes. The simulated distribution and partitioning of chlorine and bromine in these sensitivity cases are shown in SI Figure S7. Cl_*y*_ in no_Cl_emi case and Br_*y*_ in no_Br_emi case are significantly lower than their counterpart in the HAL case. Note that Br_*y*_ (34.3 pptv) in the no_Cl_emi case is only slightly lower than that in HAL case (35.6 pptv) because all bromine species are present in the gas phase and the chlorine abundance mostly impact bromine partitioning but does not imply any direct bromine source. Meanwhile, Cl_*y*_ in the no_Br_emi case (235.0 pptv) is significantly lower than that in the HAL case (334 pptv) because the presence of bromine species (HOBr, BrNO_2_ and BrNO_3_) in HAL transforms HCl to BrCl leading to evaporation of semivolatile aerosol chloride (NH_4_Cl → N*H*_3_(*g*) + HCl(g)) thereby increasing the total gaseous chlorine (Cl_*y*_). In the no_BrNO_2__uptake, the dominant species becomes BrNO_2_ instead of BrCl in the HAL case. The simulated level, distribution, and partitioning of Cl_*y*_ and Br_*y*_ in fixed_gamma and low_yield cases are similar to those in the HAL case.

**Figure 7:**
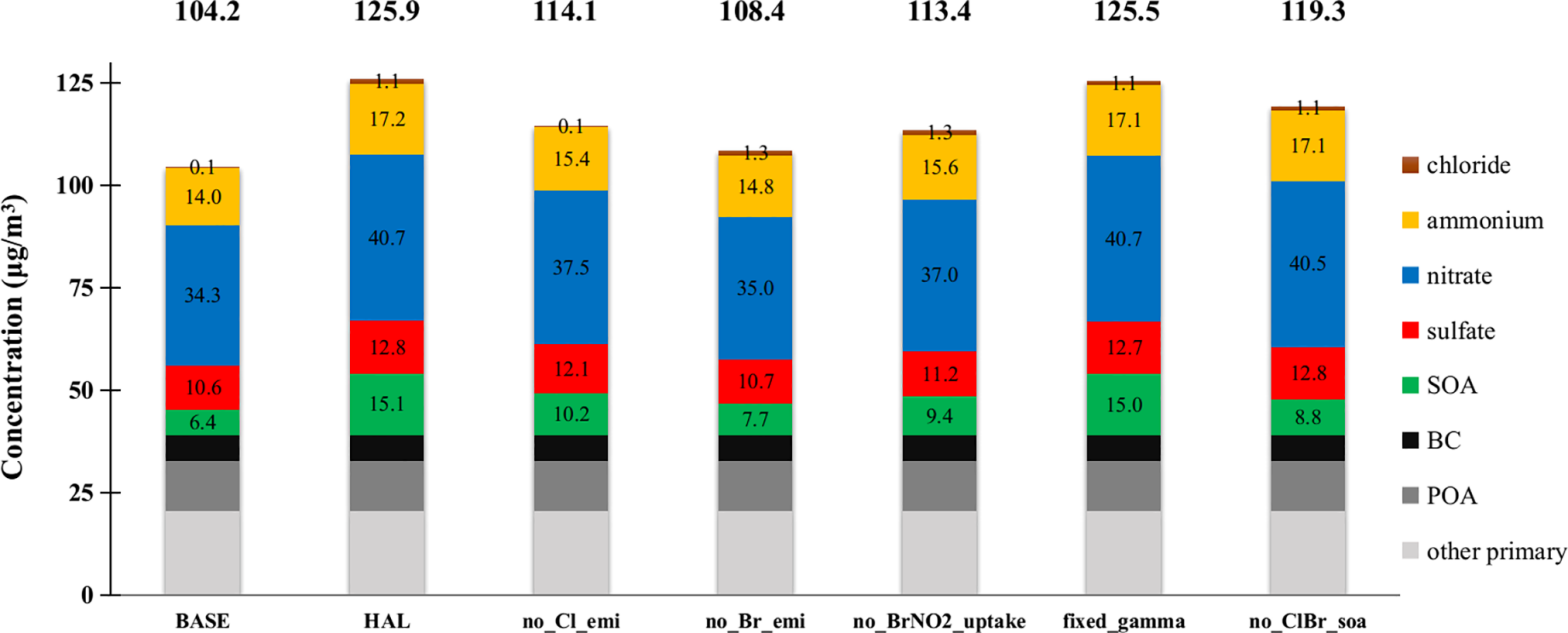
Average simulated PM_2.5_ and its components in northern China in all simulations. The number on top of each column represents the averaged PM_2.5_ concentration (μg cm^–3^) from each simulation.

Figure 7 shows the average concentration of PM_2.5_ and its components in northern China for all cases. Interestingly, anthropogenic chlorine emissions alone (no_Cl_emi v.s. HAL) increase PM_2.5_ by 11.8 μg/m^3^ (10.3%), whereas the PM_2.5_ increase by anthropogenic bromine emissions alone (no_Br_emi v.s. HAL) is 17.5 μg/m^3^ (16.1%). Our results show, for the first time, a comparable and even more important role of anthropogenic bromine emission than that of anthropogenic chlorine in producing secondary aerosols because the presence of bromine increases both the production of OH and O_3_ and the loading of the total gaseous chlorine. A recent modeling study by Wang et al.^12^ suggested an increase of 3.2 μg/m^3^ in annual average PM_2.5_ due to anthropogenic chlorine emission in China. Zhang et al.^71^ reported an enhancement of 7.5 μg/m^3^ (9.1%) in PM_2.5_ in China in winter after considering anthropogenic chlorine emission. Here we show that another halogen species (bromine) can have a similar or even larger contribution compared to that of chlorine alone.

The heterogeneous uptake of BrNO_2_ on aerosols (forming HOBr or BrCl) efficiently recycles the halogen atoms (Br and Cl) and induces a 12.5 μg/m^3^ (11.0%) increase of PM_2.5_ (no_BrNO_2__uptake v.s. HAL) which is comparable to that caused by anthropogenic chlorine. Wang et al.^72^ proposed that the role of BrNO_2_ could be vital for sustaining active bromine chemistry in low O_3_ regions. Note that BrNO_2_ was not observed above the detection limit at Wangdu site^25^ which could be due to its rapid chemical loss (photolysis and heterogeneous uptake) in the atmosphere. This is in line with SI Figure S7, which shows that if the BrNO_2_ uptake does not happen (the no_BrNO_2__uptake case), BrNO_2_ is the dominant Br_*y*_ species and would have been detected together with BrCl, HOBr, and Br_2_ at Wangdu. In light of the modeled significant effect of BrNO_2_ and its heterogeneous uptake process on Br_*y*_ partitioning and secondary aerosol production, we call for further laboratory and field studies to investigate this species and its role in aerosol formation.

Apart from BrNO_2_, other halogen species (HOCl, HOBr, ClNO_3_, and BrNO_3_) also undergo heterogeneous processes. In the HAL case, we use dynamic uptake coefficients (depending on the light intensity) to mimic the good correlation between photolysis rate and bromine levels. The small difference in bromine species (SI Figure S7) and aerosols (Figure 7) between fixed_gamma (γ = 0.1, used in previous studies^15,27^) and HAL (dynamic γ from 0.001 to >0.1) cases suggest that the halogen effects on aerosol are not sensitive to the two uptake coefficient configurations. Such a small change is observed because the heterogeneous reactions serve as recycling processes imposing larger changes to the partitioning of Br_*y*_ (e.g., larger BrCl and lower HOBr fractions in the fixed gamma case) than to the total bromine levels.

When neglecting the direct formation of SOA from Cl- and Br-initiated VOC oxidation, the SOA enhancement due to indirect halogen effect (BASE v.s. no_ClBr_SOA) is 2.4 μg/m^3^; the direct effect (no_ClBr_SOA vs. HAL) is 6.3 μg/m^3^. This result suggests that the direct halogen effect is comparable and even larger than the indirect halogen effect on SOA. Here we note that we compile the Cl-initiated SOA yield parametrizations based on the limited chamber studies and we call for the need for future chamber studies to further identify the key parameters in SOA formation from Cl- and Br-initiated reactions. Choi et al.^73^ reported that 5–10 times larger anthropogenic chlorine emissions were required to reproduce the observed chlorine species in China in winter, and such large chlorine emission could lead to an SOA increase of 0.7–3.0 μg m^–3^ in terms of direct effect and 2.5–3.0 μg m^–3^ in terms of indirect effect. The present study considers both anthropogenic chlorine (no scaling is applied) and bromine emissions and more comprehensive halogen chemistry (Cl, Br, and I) and shows a comparable increase (8.7 μg/m^3^) in SOA due to the full consideration of halogen sources and chemistry.

Despite the noted uncertainties in emissions and chemical mechanisms, this comprehensive sensitivity analysis shows the fundamental role of anthropogenic halogens in increasing haze pollution and the need to consider their emissions and chemistry in air pollution research and air quality regulation.

We demonstrate that reactive halogens (chlorine and bromine) substantially increase the loading of secondary aerosols (∼130% for SOA; ∼ 20% for sulfate, nitrate, ammonium, and PM_2.5_) in northern China during the winter season where and when haze pollution has been having severe health and visibility impacts in the past two decades. Our model exercise adopts a widely used model system with updated halogen chemistry and comprehensive halogen emissions. We find that bromine emissions, which have been previously unaccounted for in continental aerosol formation, exert a significant impact (larger than that of chlorine) on aerosols in northern China. The significant effect of bromine on aerosol formation arises for three reasons: directly via oxidation of aerosol precursors by bromine species, and indirectly by increasing the level of oxidants (OH, HO_2_, O_3_, and NO_3_), and by enhancing gaseous chlorine levels through heterogeneous reactions.

Finally, albeit our study is restricted to China, the significant range of halogen impacts on haze pollution found here is likely widespread in other continental regions with potential anthropogenic halogen emissions from coal burning, biomass burning, waste burning, etc. For instance, a recent study in India^16^ reported that chlorine increased fine aerosol via gas-particle partitioning (HCl to chloride and NH_3_ to ammonium) and water uptake on existing aerosol, implying that the role of halogens in the chemical production of secondary aerosol could also be important in South Asia as well as other continental regions where anthropogenic halogens (chlorine and bromine) emissions and chemistry have also been shown to impact gas-phase atmospheric compositions^74,75^

We call for more studies to improve the understanding of emission, chemistry, and impacts of anthropogenic reactive halogens, for example, direct measurement of bromine emission factors of various potential sources, laboratory experiments on Cl- and Br-initiated SOA formation, and field observations of the multiphase processes of halogen species, etc.

## Supporting Information Available

Processes related to secondary aerosol formation in standard WRF-Chem, Chlorine-initiated SOA yield parametrization, WRF-Chem sensitivity design, and WRF-Chem model evaluation (PDF)
(XLSX)
