# Supplementary material for: Halogens Enhance Haze Pollution in China
Source: Environ Sci Technol. 2021 Sep 30;55(20):13625–37. doi: 10.1021/acs.est.1c01949 (PMC8529710; doi:10.1021/acs.est.1c01949)
Supplement: Supplementary file 1 — es1c01949_si_001.pdf [file es1c01949_si_001.pdf]

Supplementary Materials for

Halogens enhance haze pollution in China

Qinyi Li[1,‡], Xiao Fu[2,3,‡], Xiang Peng[2], Weihao Wang[2,#], Alba Badia[4], Rafael P. Fernandez[1,5], Carlos A. Cuevas[1], Yujing Mu[6], Jianmin Chen[7], Jose L. Jimenez[8], Tao Wang[2,*], and Alfonso Saiz-Lopez[1,*]

[1] Department of Atmospheric Chemistry and Climate, Institute of Physical Chemistry Rocasolano, CSIC, Madrid 28006, Spain

[2] Department of Civil and Environmental Engineering, The Hong Kong Polytechnic University, Hong Kong 999077, China

[3] Institute of Environment and Ecology, Tsinghua Shenzhen International Graduate School, Tsinghua University, Shenzhen 518055, China

[4] Institute of Environmental Science and Technology (ICTA), Universitat Autònoma de Barcelona (UAB), Barcelona 08193, Spain

[5] Institute for Interdisciplinary Science (ICB), National Research Council (CONICET), FCEN-UNCuyo, Mendoza M5502JMA, Argentina

[6] Research Center for Eco-Environmental Sciences, Chinese Academy of Sciences, Beijing 100085, China

[7] Department of Environmental Science and Engineering, Fudan University, Institute of Atmospheric Sciences, Shanghai 200433, China

[8] Cooperative Institute for Research in Environmental Sciences and Department of Chemistry, University of Colorado, Boulder, CO 80309, USA

[‡] These authors contributed equally.

[#] Present address: Hangzhou PuYu Technology Development Co. Ltd, Hangzhou 311305, China.

[*] Correspondence to Alfonso Saiz-Lopez (a.saiz@csic.es) and Tao Wang (tao.wang@polyu.hk)

Contents:

Supplementary Text S1 to S4

S1. Processes related to secondary aerosol formation in standard WRF-Chem

S2. Chlorine-initiated SOA yield parameterization

S3. WRF-Chem sensitivity design

S4. WRF-Chem model evaluation

Supplementary Figures S1 to S10

Supplementary Tables S1 to S4

Supplementary References

Supplementary Text

S1. Processes related to secondary aerosol formation in standard WRF-Chem

Here we briefly introduce the processes, relevant to the formation of secondary aerosols, in the standard WRF-Chem. The new reactions added to WRF-Chem in the present work are described in the Materials and Methods in the main text.

(1) In the standard WRF-Chem (v3.7.1), the gas phase scheme (MOZART) has been described in detail in Emmons et al. [1] and the formation pathways of secondary aerosols in the MOSAIC aerosol scheme have been documented in Zaveri et al. [2], Shrivastava et al. [3], and Knote et al. [4]:

a. SOA. The formation of SOA is initiated with the oxidation of VOCs by oxidants (OH, O_3, and NO_3). While OH radical mostly determines the VOC oxidation during the daytime, O_3 and NO_3 are the vital oxidants during the night. The processes related to glyoxal also contribute to the loading of SOA, including the oligomerization reactions, surface uptake on aerosols, ammonium-catalyzed reaction, and OH reaction.

$$\mathrm{VOC + OH/O_3/NO_3 \rightarrow SOA} \quad \text{(RS1)}$$

$$\mathrm{GLY \rightarrow SOA} \quad \text{(RS2)}$$

b. Sulfate. Sulfate aerosol is formed through the reaction of SO_2 with OH (gas phase) as well as O_3, H_2O_2, and O_2-Fe-Mn (aqueous phase).

$$\mathrm{SO_2+OH \rightarrow sulfate} \quad \text{(RS3)}$$

$$\mathrm{SO_2+O_3/H_2O_2(aq) \rightarrow sulfate} \quad \text{(RS4)}$$

$$\mathrm{SO_2+O_2\ (Fe,Mn;\ aq) \rightarrow sulfate} \quad \text{(RS5)}$$

c.Nitrate. Nitrate aerosol is in thermodynamic equilibrium with nitric acid gas which is produced via the reactions of NO_2 with OH (gas phase) and N_2O_5 hydrolysis (multiphase). The formation channels of nitrate aerosol with sodium, magnesium, and potassium, etc. are not considered.

$$\mathrm{NO_2+OH \rightarrow HNO_3} \quad \text{(RS6)}$$

$$\mathrm{N_2O_5+H_2O(aq) \rightarrow 2HNO_3(aq)} \quad \text{(RS7)}$$

d. Ammonium. Ammonium aerosol is formed from the uptake of ammonia gas (NH_3) to neutralize sulfuric, nitric, and hydrochloric acids in the aerosol, in equilibrium with the gas-phase species.

$$\mathrm{NH_3+H_2SO_4 \rightarrow (NH_4)_2SO_4} \quad \text{(RS8)}$$

$$\mathrm{NH_3+HNO_3 \leftrightarrow NH_4NO_3} \quad \text{(RS9)}$$

$$\mathrm{NH_3+HCl \leftrightarrow NH_4Cl} \quad \text{(RS10)}$$

(2) Archer-Nicholls et al. [5] replaced the N_2O_5 hydrolysis reaction with N_2O_5 uptake on the chloride-containing aerosol surface which consumes chloride and produces nitrate.

$$\mathrm{N_2O_5+H_2O(aq)+Cl^-(aq) \rightarrow NO_3^-(aq)+ClNO_2} \quad \text{(RS11)}$$

(3) Badia et al. [6] incorporated halogen chemistry in the MOSAIC aerosol scheme and the MOZART gas-phase scheme. These added halogen reactions generally induce indirect effect on secondary aerosol:

a. halogen chemistry substantially perturbs the levels of oxidants (OH, O_3, and NO_3) and therefore affects the production of SOA, sulfate, nitrate, and ammonium.

$$VOC+Cl \rightarrow RO_2+HO_2 \quad \text{(RS12)}$$

$$X+O_3 \rightarrow XO+O_2 \text{ (X=Cl, Br, I)} \quad \text{(RS13)}$$

$$XO+HO_2 \rightarrow HOX+O_2 \quad \text{(RS14)}$$

$$HOX+h\nu \rightarrow OH+X \quad \text{(RS15)}$$

$$XO + NO \rightarrow X+NO_2 \quad \text{(RS16)}$$

$$XO + NO_2 \rightarrow XONO_2 \quad \text{(RS17)}$$

$$NO_2 + O_3 \rightarrow NO_3 \quad \text{(RS18)}$$

b. the heterogeneous uptake of halogen nitrates on the aerosol surface to form nitric acid which is in equilibrium with nitrate aerosol.

$$XO+NO_2 \rightarrow XONO_2 \quad \text{(RS19)}$$

$$XONO_2 +H_2O \text{ (aq)} \rightarrow HNO_3+HOX \quad \text{(RS20)}$$

$$XONO_2+HY \rightarrow HNO_3+XY \text{ (Y=Cl, Br, I)} \quad \text{(RS21)}$$

c. the multiphase halogen chemistry affects the abundance of HCl and chloride aerosol (therefore affecting the budget of ammonium).

$$VOC+Cl \rightarrow HCl+products \quad \text{(RS22)}$$

$$HOX+HCl \rightarrow XCl +H_2O \quad \text{(RS23)}$$

$$XONO_2+HCl \rightarrow XCl+HNO_3 \quad \text{(RS24)}$$

d. the effects of halogen on sulfate and nitrate further impact the partitioning of NH_3 to the aerosol to form ammonium.

S2. Chlorine-initiated SOA yield parameterization

The existing SOA yield parameterizations in chemical transport models all follow Odum et al. [7] in which the observed total SOA yield (also known as aerosol mass fraction, AMF) in the chamber studies can be used to derive the parameterization using the following non-linear equation.

$$AMF = \sum_1^n \frac{\alpha_1}{(1+\frac{C_1^*}{C_{OA}})} \quad \text{(RS25)}$$

in which α is the mass yield, C* is the effective saturation concentration, and C_{OA} is the organic aerosol loading (usually the formed SOA amount in chamber studies) of each gaseous product.

Traditionally, SOA yield parameterizations adopted two-products (i.e. n=2 in RS25) scheme and used the chamber studies to determine the four free parameters (α_1, C_1^*, α_1, C_2^*) in the parameterization. Recently, it has been proposed that the two-products scheme has some limitations in chemical

transport model application (e.g., Murphy and Pandis [8]), including (1) two products are required for each VOC species; (2) no multigeneration reactions can be applied after the first step of VOCs oxidation; (3) the range of organic aerosol loading is limited to two bins. In the standard WRF-Chem model (v3.7.1) MOZART-MOSAIC-VBS mechanism, the formation of SOA is treated with the VBS framework [8-10]. In this framework, the products of VOCs oxidation are represented by four condensable gaseous products (CGP) with fixed C^* of 1, 10, 100, and 1000 μg m^{-3}, respectively, and mass yields of α_1, α_2, α_3, and α_4, respectively (RS26). The advantages of VBS framework include (1) four CGPs are used to represent all VOC oxidation products; (2) there is still four free parameters as the two-products scheme; (3) the VBS framework extends the organic aerosol range to four orders of magnitude and a larger range is possible; (4) further gaseous oxidation of CGPs can be achieved by oxidizing CGPs with higher C^* to form those with lower C^*.

$$\text{VOC} + \text{oxidant} \rightarrow \alpha_1\text{CGP}_1 + \alpha_2\text{CGP}_2 + \alpha_3\text{CGP}_3 + \alpha_4\text{CGP}_4 \qquad \text{(RS26)}$$

Here we used the available chamber data of Cl-initiated SOA yield experiment data (AMF and C_{OA}) to derive the values of α. The fitting curves are shown in Figure S1 and the derived α values in Table S2. Here we briefly describe the chamber data used in our study. Isoprene observations are from Wang and Hildebrandt Ruiz [11] and we also plotted their parameterization using a VBS of 0.1, 1, 10, 100 μg/m^3 as a reference (isoprene_fit_WH in Figure S1). The chamber data of α-pinene, β-pinene, and limonene are from Cai and Griffin [12], in which limonene high level data are used as the high yield or low-NO_x parameterization and limonene low level data as low yield or high NO_x parameterization. The chamber data of α-pinene_obs_O2013 is from Ofner et al. [13]. Alkane data are from Wang and Hildebrandt Ruiz [14]. Toluene_low_NO_x and Toluene_high_NO_x are from Dhulipala et al. [15]. Toluene obs_C2008 are from Cai et al. [16].

There is not enough data for isoprene, β-pinene, and alkane to distinguish between the high- and low-NO_x conditions, so the same parameters are applied for both conditions. For α-pinene and limonene, the parameterizations with lower yield are used for high-NO_x parameterization, and those with higher yield as low-NO_x condition because SOA yield in low-NO_x condition is usually larger than that in high-NO_x conditions [10]. For toluene, the fitted parameters using high NO_x data are even higher than those using the low NO_x data, which is contrary to VBS framework [10], so the parameters of low NO_x data are used in the study for both conditions. Future chamber studies are desired to improve the representation of Cl-initiated SOA yield parameterization in the chemical transport models.

S3. WRF-Chem sensitivity design

Here we describe the sensitivity simulations:

(1) no_Cl_emi case does not have anthropogenic chlorine emission in China but has everything else in HAL case; the changes from no_Cl_emi to HAL represent the effect of anthropogenic chlorine emissions.

(2) no_Br_emi scenario excludes the anthropogenic bromine emission over China; so the difference between no_Br_emi and HAL represents the effect of anthropogenic bromine emissions.

(3) no_$BrNO_2$_uptake simulation deactivates the two heterogeneous reactions of $BrNO_2$ on the aerosol surface; therefore, the changes from no_$BrNO_2$_uptake to HAL are the influence of the heterogeneous uptake process of $BrNO_2$.

(4) the fixed_gamma case uses a constant uptake coefficient for the bromine heterogeneous reactions; the effects of halogen chemistry with a fixed gamma are shown by the changes from BASE to fixed_gamma.

(5) the no_ClBr_SOA simulation neglects the direct formation of SOA from the Cl and Br-initiated VOCs oxidations; so the comparison of the simulated SOA between BASE and no_ClBr_SOA demonstrates the indirect effect of halogens on SOA, and that between no_ClBR_SOA and HAL is the direct effect of halogens on SOA.

S4. WRF-Chem model evaluation

We used the field measurement data during the Wangdu campaign [17], the only available dataset of reactive bromine species in China, to validate the WRF-Chem simulation (HAL scenario) for reactive halogen species (Figure S8). WRF-Chem generally reproduces the magnitude and variation of BrCl, the dominant bromine and chlorine species on most days, with the simulation average of 46.9 pptv, the observation average of 64.7 pptv, and normalized mean bias (NMB) of -27.5%. The model underestimates HOBr (12.6 pptv, simulated average; 33.6 pptv, observed average; NMB of -62.5%) and overestimates Br_2 (5.8 pptv, simulated average; 3.2 pptv, observed average; NMB of +81.3%). The simulated level and temporal variation of $ClNO_2$ are in line with the observation in most days with a simulation average of 76.9 pptv, the observation average of 102.9 pptv, and NMB of -25.3%. Notably, WRF-Chem also captures the abrupt increase of $ClNO_2$ on Dec 14-15 and Dec 27-28. The underestimation may partly result from the underestimation of particle chloride (1.6 μg m^{-3}, simulated average; 6.5 μg m^{-3}, observed average; NMB of -75.4%) (Figure S10).

A very recent observation of HBr in Beijing (in northern China) in winter of 2019 reported a mixing ratio of ~2 pptv to ~50 pptv [18]. Our simulation predicted a mixing ratio range of <1 pptv to 12 pptv at Beijing in the winter of 2017, which is within the range of the observation although with some underestimation. Overall, the current setup of the model is able to reproduce the level and the variation of the halogen species but with some underestimation, therefore, the impact of chlorine and bromine in the present study is considered as the lower limit in this region.

A few global and regional modeling works have estimated the levels of halogen species (mostly chlorine) in China. The estimations involving anthropogenic chlorine emissions [19-23] showed a similar chlorine level as in this study, while the estimations without anthropogenic chlorine emissions [24] showed a much lower level. Previous studies [25-29] have simulated a very limited level of inorganic bromine species (<1 pptv) in China (and other continental areas) due to the lack of anthropogenic inorganic bromine sources. The levels of halogen species (especially the bromine) and their impacts on oxidation and aerosol production over the continental area in these previous studies are expected to be underrated significantly.

Our WRF-Chem model successfully reproduces the routine gas-phase pollutants and variables (Figure S 9). The simulated photolysis rate of NO_2 (J_{NO2}, an indicator of the solar radiation) in WRF-Chem model (average value hereafter, 0.001 s^{-1}) is consistent with the measured values (0.00095 s^{-1}). The variation of J_{NO2} magnitude is very similar to the variation of the observed BrCl variability, therefore, we decided to use light-dependent uptake coefficients for the bromine species heterogeneous uptake processes in the HAL case and a fixed_gamma case to examine the sensitivity to this treatment (Supplementary Text S3; Table S4). Our model reproduced well the magnitude and variation of SO_2

and NO_2, with the NMB of +4.8% and -3.0%. The simulated O_3 (11.4 ppbv) is slightly larger than the observation (8.2 ppbv) with an NMB of +39.0% but with a similar diurnal cycle. The modeled O_x (O_3+NO_2, 40.5 ppbv) is very similar to the observed values (38.2 ppbv) with an NMB being only +6.1%. The WRF-Chem model also captures the magnitude and the general variation of the total reactive nitrogen (NO_y) with an average of 110.5 ppbv in HAL case and that of 118.1 ppbv for observation and an NMB of -6.4%. Our model is able to reproduce the level of the main precursors of oxidants, HONO and H_2O_2, with a simulation average of 2378.8 pptv and 209.8 pptv, respectively, while the observation average is 2389.5 pptv and 209.3 pptv, respectively, and the NMB being -0.4% and 0.2%, respectively.

As to the aerosol components (shown in Figure S10), the predicted temporal variations of $PM_{2.5}$ track the measurements well. The average simulated $PM_{2.5}$ in BASE case is 116.8 μg m^{-3}, and that in HAL is 140.1 μg m^{-3}, while the average observed $PM_{2.5}$ is 175.1 μg m^{-3}, suggesting that the addition of halogen chemistry decreases the NMB from -33.3% in the BASE case to -20.0% in the HAL case. The remaining underestimation of $PM_{2.5}$ is possibly due to the underestimation of primary aerosol, which may result from the missing local emission sources in the current emission inventory or the missing chemical mechanisms of secondary aerosol formation.

The predicted sulfate concentration (13.7 μg m^{-3}) is very close to the observation (12.3 μg m^{-3}) with an NMB of only +11.4%. The WRF-Chem model results of sulfate also track well the observation with a correlation coefficient of 0.58.

The simulated concentration of nitrate aerosol (40.6 μg/m^3) is overestimated (26.6 μg/m^3 or 0.39 μmol/m^3 higher) compared to the observation (14.0 μg/m^3). The modeled ammonium (17.7 μg/m^3) is also larger (6.6 μg/m^3 or 0.37 μmol/m^3 higher) than the observed concentration (11.1μg/m^3). The overprediction of nitrate is very similar to that of ammonium (in molar density, μmol/m^3) and the simulated NH_3 is underestimated by 34.4% (Figure S9), implying that such overestimation of nitrate and ammonium and underestimation of NH_3 are likely due to the uncertainty of the thermodynamic equilibrium parameterization in WRF-Chem which overestimates the partitioning of gaseous NH_3 and HNO_3 to NH_4NO_3 aerosol. Note that no observation of HNO_3 is available during the Wangdu campaign. The fact that our model reproduces well the level of NO_y at Wangdu site (Figure S9) also suggests that the overestimation of nitrate aerosol is more likely a partitioning issue. Ianniello et al. [30] reported observations of HNO_3, NH_3, nitrate, and ammonium in Northern China and they found that the levels of HNO_3 and NH_3 in winter (as in our study) are much larger than the prediction of the gas-particle theory, implying that the calculated losses of NH_3 and HNO_3 (and formation of ammonium nitrate) are too fast compared to the observation in winter in northern China. Similar overestimation of nitrate and ammonium has been reported by another WRF-Chem study [31] in this region during some days in their simulation. Such uncertainty of the thermodynamic simulation in WRF-Chem has also been proposed to affect the multiphase chemistry simulation [32]. Note that the halogen impacts on nitrate and ammonium aerosol remain the same (~30% at Wangdu site) when we arbitrarily decreased the formation rate of the ammonium nitrate and the loss rate of HNO_3 and NH_3 in a test run. Apart from the gas-particle partitioning, we also consider other possibilities of the overestimated nitrate and ammonium. Shah et al. [33] suggested that updating the dry deposition parameterization of HNO_3 (Wesely et al. [34]) helped to reduce the over-predicted nitrate in the GEOS-Chem model. However, in the current study (applying the same dry deposition scheme and the same adjustment following Shah et al. [33]), the modeled HNO_3 is very low (<1 μg/m^3) compared to the nitrate aerosol (~40 μg/m^3), therefore, modification of HNO_3 dry deposition does not induce a noticeable effect on nitrate aerosol. We also cut the emission of NO_x in a test case; while such an attempt reduced nitrate aerosol, it

jeopardized the well-reproduced NO_y and O_3. We also conducted a test case via reducing NH_3 emission by 30% which resulted in only ~10% reduction in nitrate and ammonium aerosol but ~50% decrease in NH_3 gas, highlighting the nonlinear nature of the formation of ammonium nitrate aerosol to the changes in NH_3 sources. A recent paper by Liu et al. [35] provided a more detailed analysis on this matter. To summarize, (a) gas-particle partitioning is most likely the cause of the overestimated nitrate and ammonium in our study; (b) detailed investigation on this issue requires future studies; (c) arbitrarily tuning the partitioning does not change the halogen impact on nitrate and ammonium; (d) in the current work, we focus on the relative impact on secondary aerosols induced by halogens.

The model captures the variation trend of the observed SOA. Compared with the BASE case (3.5 μg m^{-3}), the addition of halogens significantly increases SOA by 194% to 10.3 μg m^{-3} at Wangdu site and helps to close the gap between the simulation and observation (16.6 μg m^{-3}) The underestimation on Dec 22-23 and Dec 29-30 may result from the fact that current chemical mechanisms are still unable to fully explain the oxidation of VOC and the ageing process of organic aerosol [36,37]. Insufficient information, e.g., household sources [38], used in developing the VOCs emission inventory could also affect the SOA simulation.

The observed chloride concentrations at Wangdu site were high, with an hourly peak of 32 μg/m^3, comparable with the concentrations of nitrate and ammonium. Our simulation underestimated such high chloride, which may be affected by specific emissions at local scale, which cannot be represented by regional emissions.

The prediction of a primary aerosol, black carbon (BC), shows a good agreement in magnitude and variation with the observation. The average simulated concentration in HAL is 9.5 μg m^{-3}, and the average observation is 8.6 μg m^{-3}, and the NMB is +10.5%.

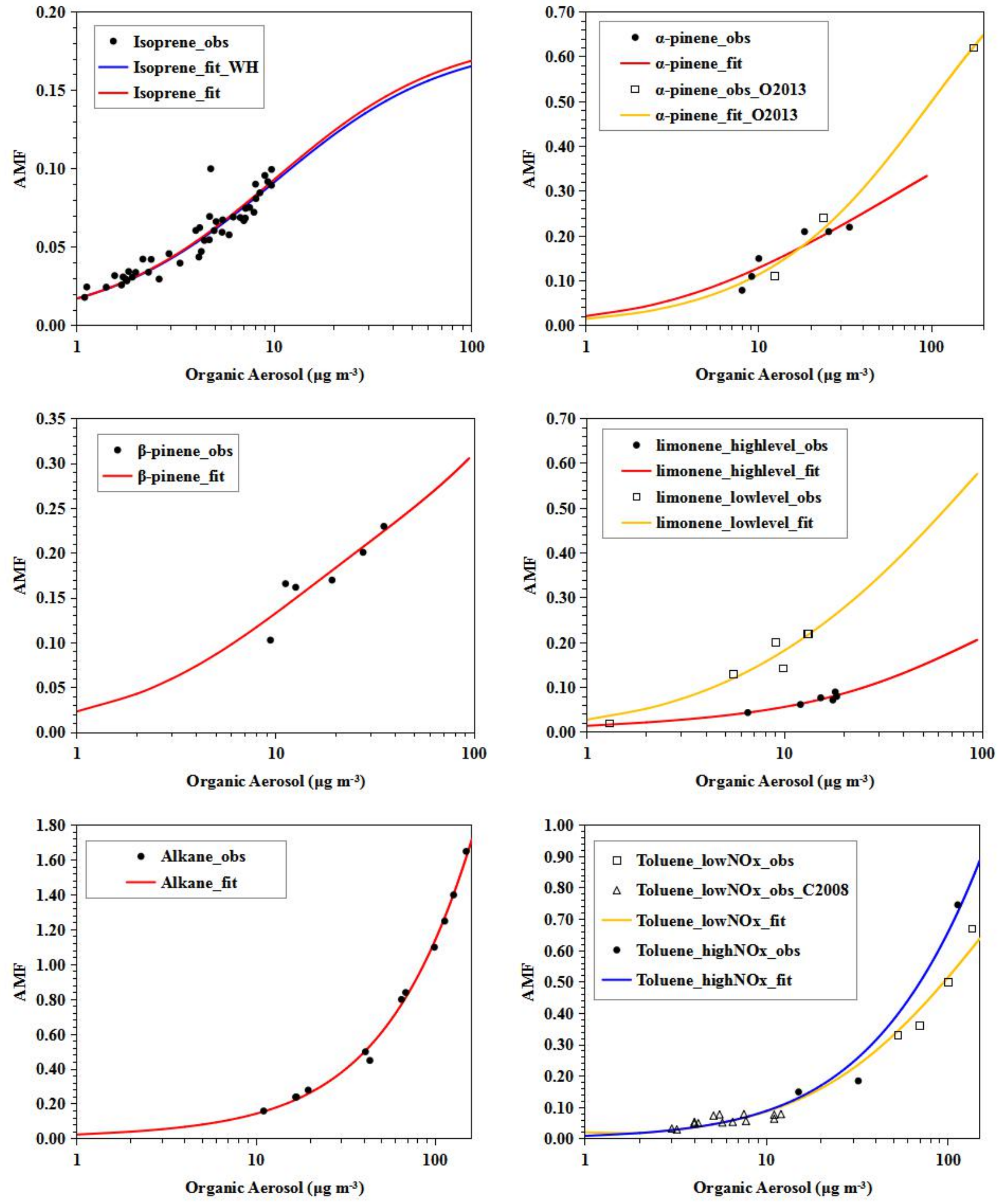

Figure S1 | Observation of SOA aerosol mass fraction (AMF; scatter) and the fitting SOA yield parameterization (line) for isoprene, α-pinene, β-pinene, limonene, alkane, and toluene. The coefficients of the fitted parameterization are shown in Table S2.

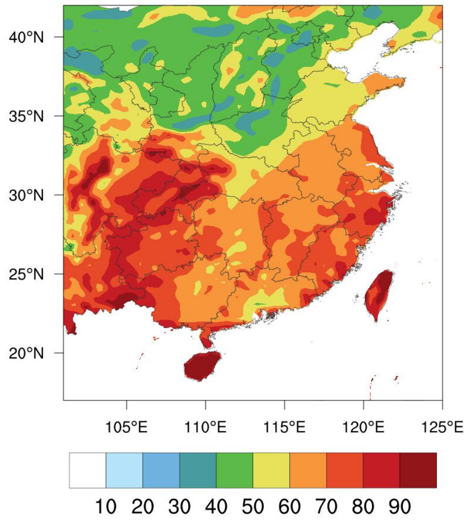

Figure S2 | Average simulated relative humidity (%) in the HAL case at the surface in China.

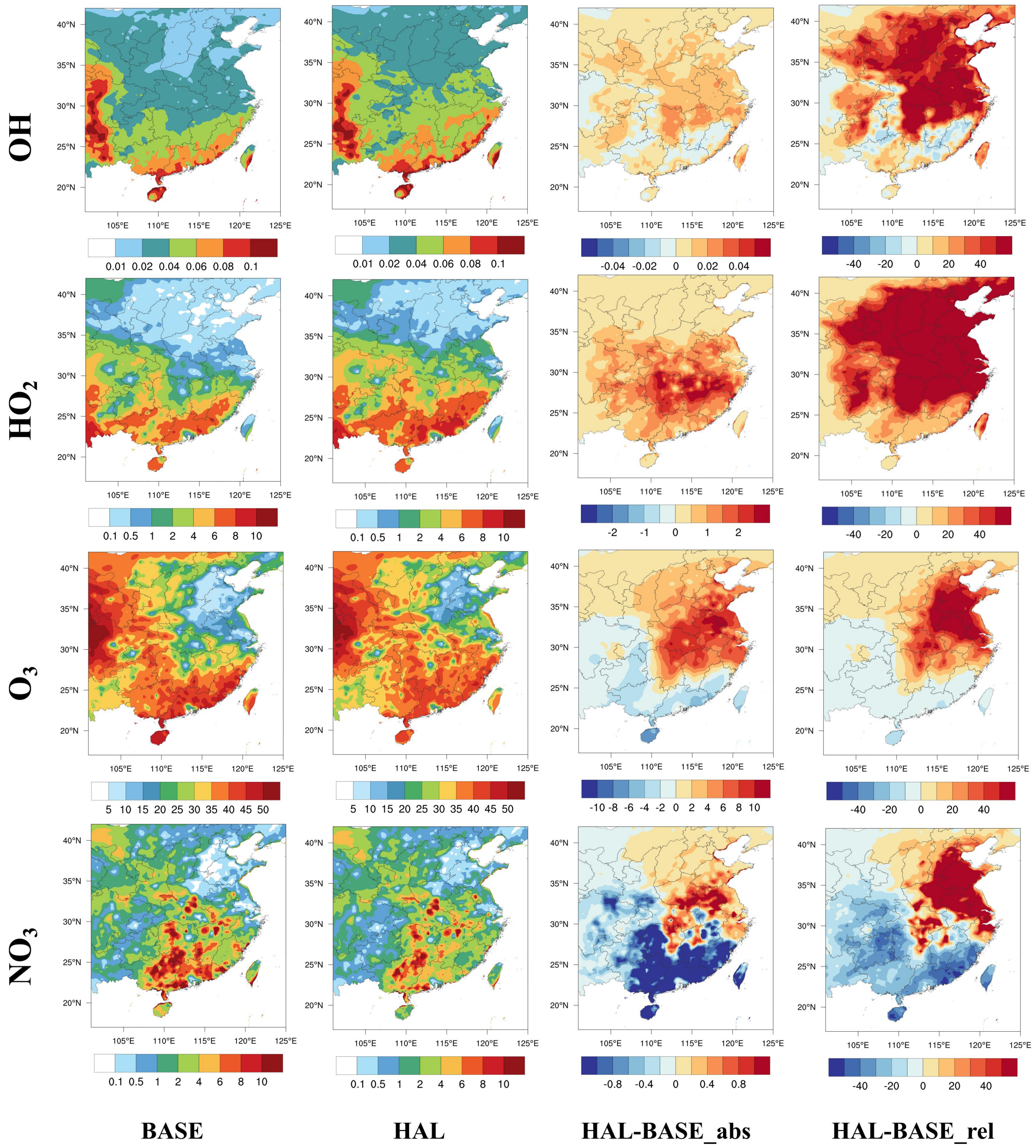

Figure S3 | Average simulated level of oxidants (OH, pptv; HO_2, pptv; O_3, ppbv; and NO_3, pptv) in BASE and HAL cases at the surface in China. The absolute changes (pptv for OH, HO_2, and NO_3, and ppbv for O_3) and relative changes (%) are also presented.

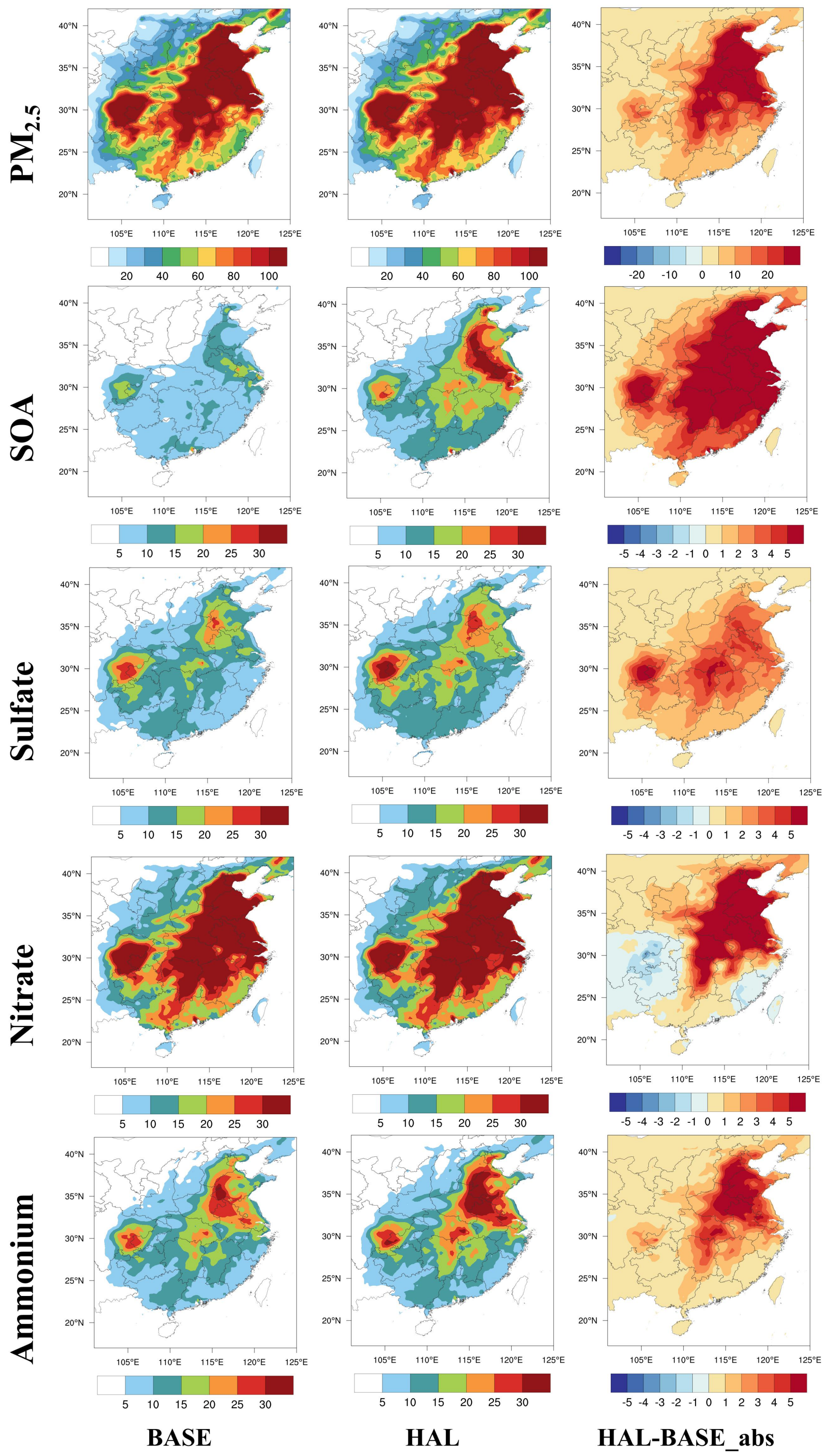

Figure S4 | Average simulated level of fine aerosol (μg m^{-3}) and its major composition (SOA, sulfate, nitrate, ammonium; μg m^{-3}) in BASE (left column) and HAL (middle column) cases at the surface in China. The right column shows the absolute changes in μg m^{-3}.

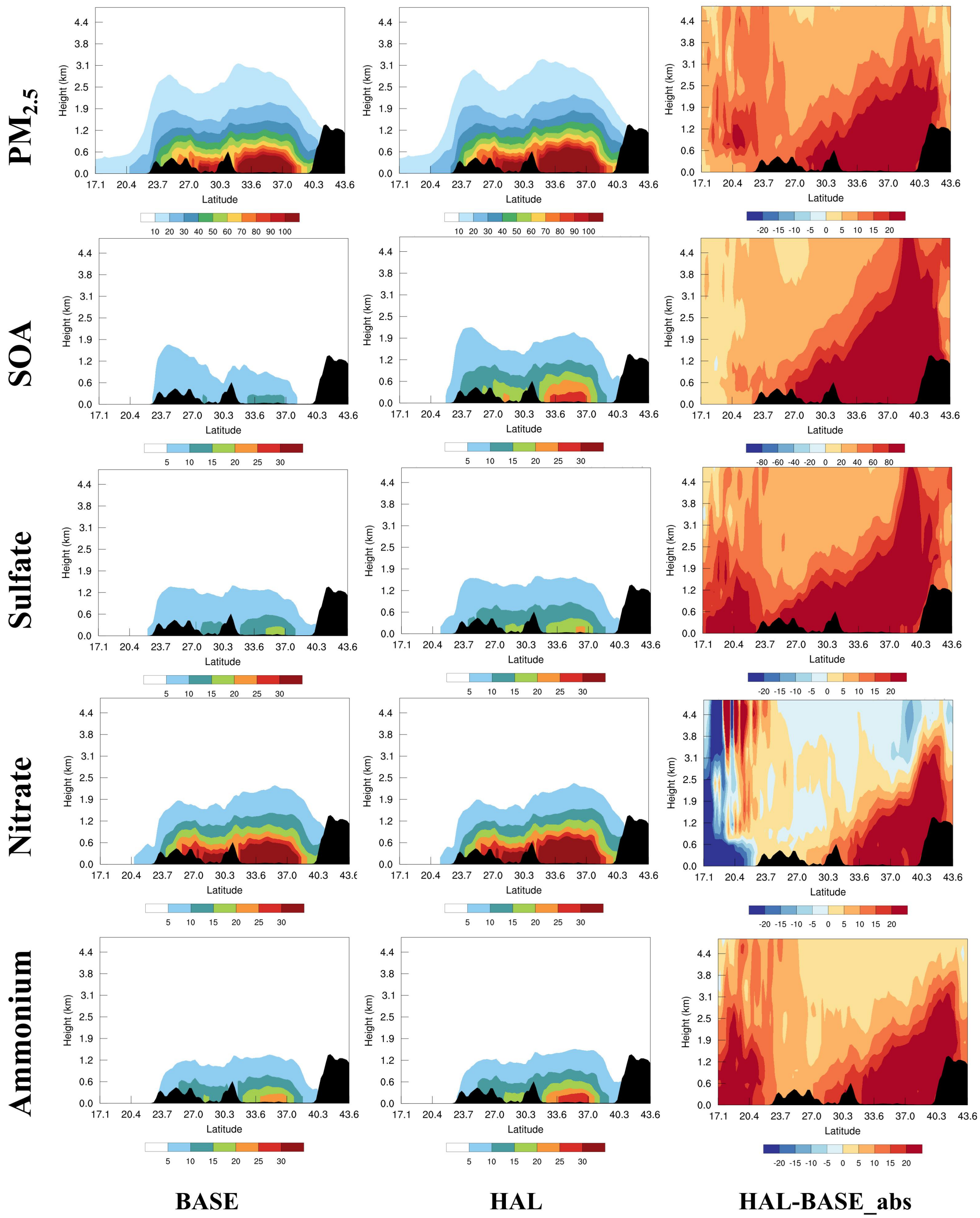

Figure S5 | Average simulated level of fine aerosol (μg m^{-3}) and its major composition (SOA, sulfate, nitrate, ammonium; μg m^{-3}) in BASE (left) and HAL (middle) cases in the south-north cross-section in China (see location in Figure 1). The black shaded area is the mountainous region. The absolute changes (μg m^{-3}) are also presented on the right column.

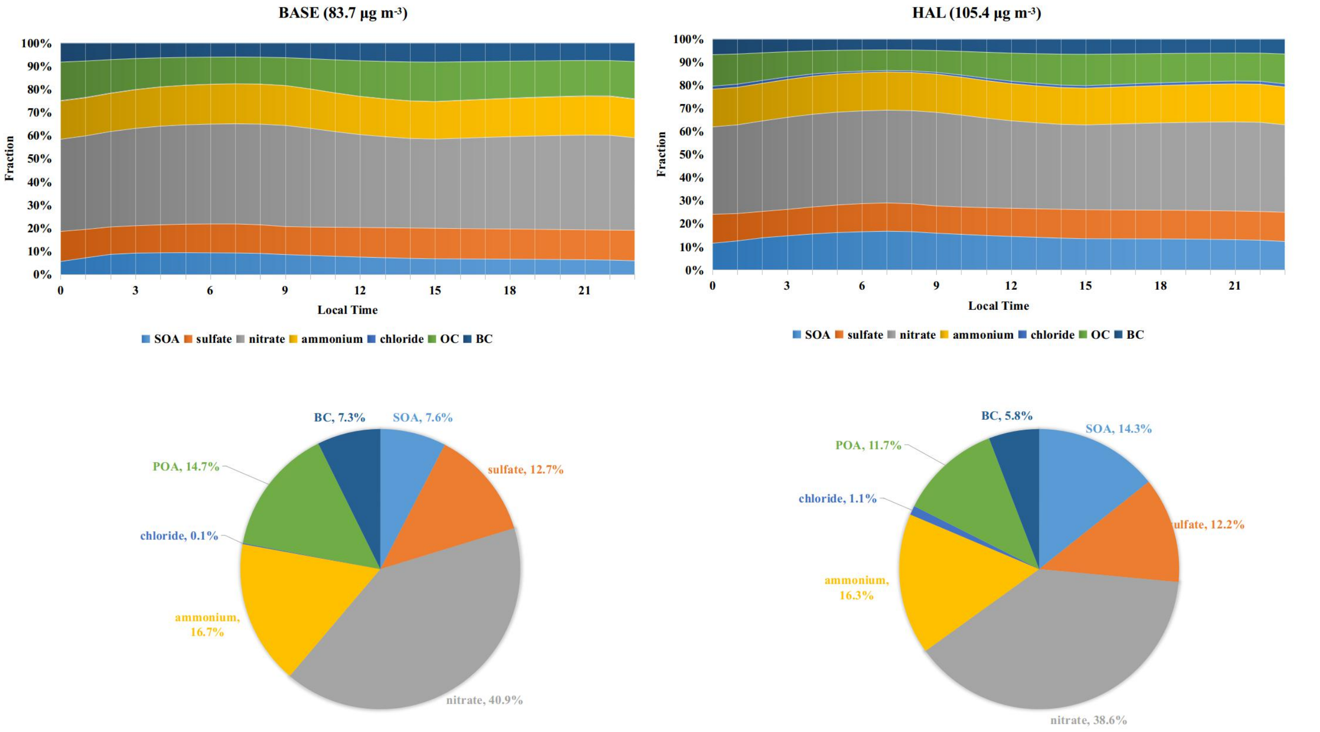

Figure S6 | Average diurnal variation of aerosol composition in northern China in BASE (upper-left) and HAL (upper-right) cases. Average simulated composition of fine aerosol at the surface in northern China in BASE (lower-left) and HAL (lower-right) scenarios. Note that only the speciated components of $PM_{2.5}$ are shown in this figure. Note that the simulated levels of primary aerosols (POA and BC) are the same in BASE and HAL cases; the levels of secondary aerosols are higher in the HAL cases than those in the BASE case; resulting lower fraction (%) of primary aerosols in the HAL case.

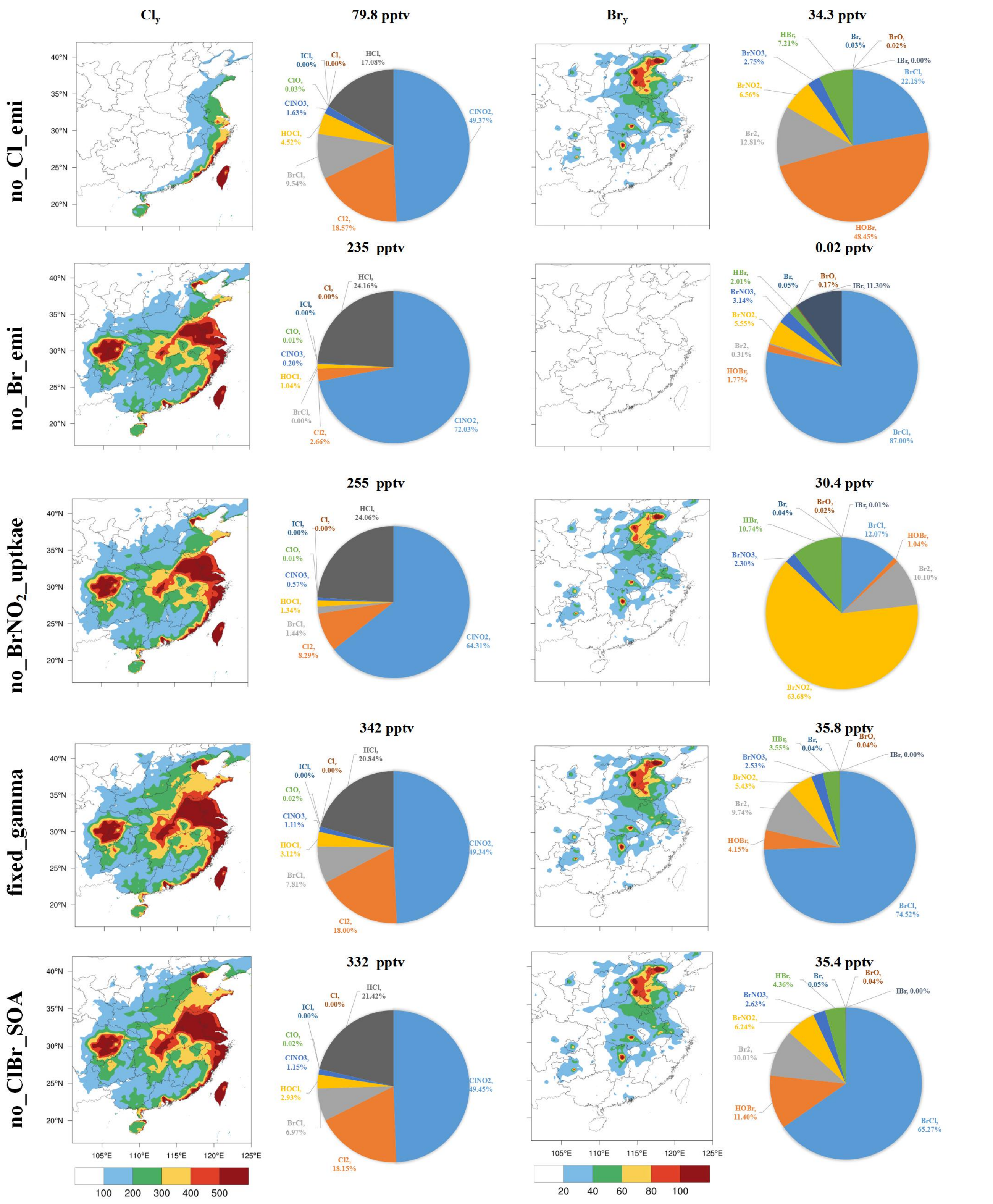

Figure S7 | Average simulated Cl_y (pptv) and Br_y (pptv) at the surface in the five sensitivity cases and their average partitioning in northern China. The description of the difference in these cases is shown in Table S4.

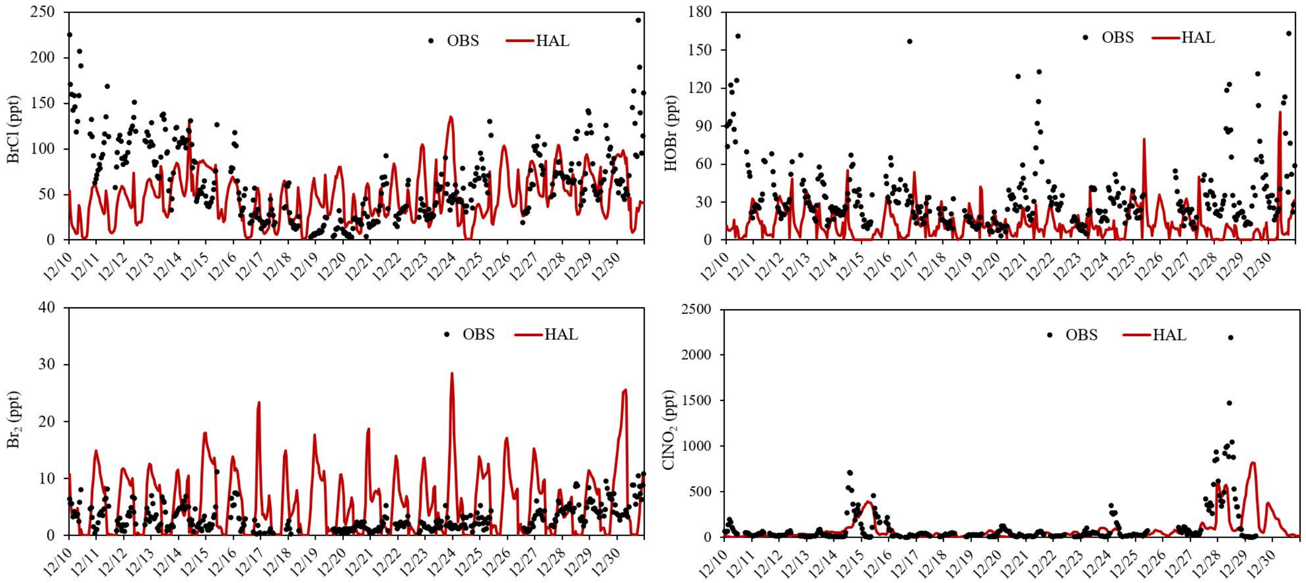

Figure S8 | Simulated and observed hourly variations of BrCl (pptv), HOBr (pptv), Br_2 (pptv), and $ClNO_2$ (pptv) during the comprehensive campaign in Wangdu in Dec 2017.

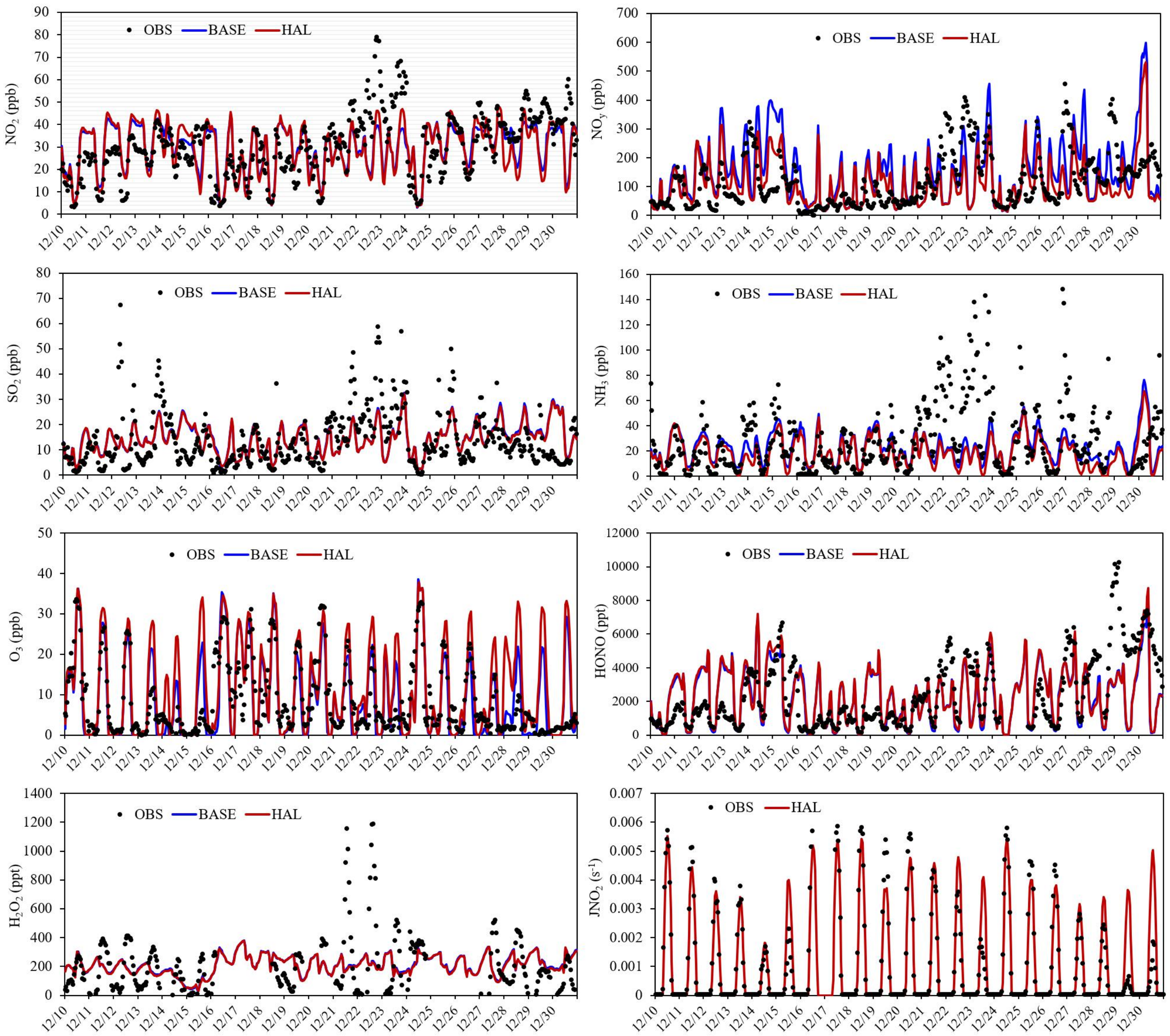

Figure S9 | Simulated and observed hourly variations of NO_2 (ppbv), NOy (ppbv), SO_2 (ppbv), NH_3 (ppbv), O_3 (ppbv), HONO (pptv), H_2O_2 (pptv), and JNO_2 (s^{-1}) during the comprehensive campaign in Wangdu in Dec 2017.

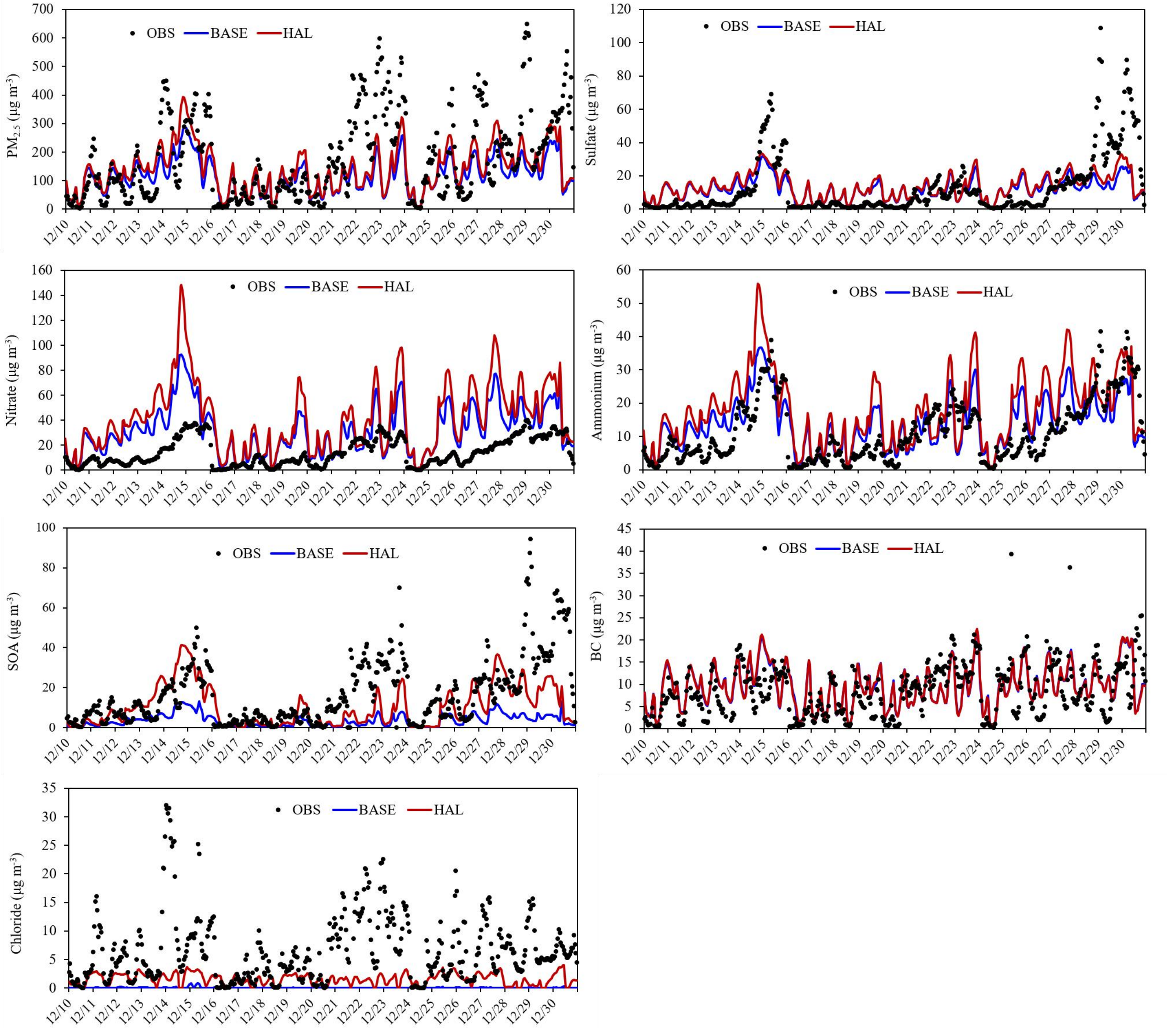

Figure S10 | Simulated and observed hourly variations of $PM_{2.5}$, sulfate, nitrate, ammonium, SOA, black carbon, and chloride during the comprehensive campaign in Wangdu in Dec 2017. All units are $\mu g\ m^{-3}$.

Table S1 | Bromine release rates for different combustion technology and process

Sector	Technology	Br release rate	Reference
Power plants/ Industrial boiler	pulverized coal boiler<br>circulating fluidized bed<br>stoker furnace	98%	James et al. [39]; Klein [40]; Klika et al. [41]; Peng et al. [42]; Song et al. [43]; Wang et al. [44]
Industrial process	sinter/pelletizing	98%	The same value with industrial boilers was set.
	cement kilin	30%	Referring to the SO_2 release rates in Lei et al. [45].
	lime kilin	70%	Referring to the SO_2 release rates in Lei et al. [45].
	brick kilin	98%	The same value with industrial boilers was set.
Residential burning	stoker furnace	98%	The same value with industrial boilers was set.
	stove	80%	Peng et al. [46].

Table S2 | Chlorine-induced SOA mass yield parameterization

VOC	High-NO_x Parameterization				Low-NO_x Parameterization			
	1*	10*	100*	1000*	1*	10*	100*	1000*
Isoprene	0.0012	0.1846	0.0000	0.0000	0.0012	0.1846	0.0000	0.0000
α-pinene	0.0000	0.2013	0.3145	0.0000	0.0000	0.0703	0.8727	0.0000
β-pinene	0.0000	0.2473	0.0000	0.9593	0.0000	0.2473	0.0000	0.9593
Limonene	0.0195	0.0119	0.3636	0.0000	0.0000	0.2264	0.7678	0.0000
Alkane	0.0251	0.0000	0.0000	12.29	0.0251	0.0000	0.0000	12.29
Toluene	0.0309	0.0000	0.0000	5.2893	0.0309	0.0000	0.0000	5.2893

* The numbers 1, 10, 100, 1000 represent the saturation concentrations (μg m^{-3}) of the surrogate species.

Table S3 | Physical and chemical modules adopted in WRF-Chem

Category	Options
Shortwave radiation	RRTMG shortwave radiation scheme (Iacono et al. [47])
Longwave radiation	RRTMG longwave radiation scheme (Iacono et al. [47])
Land-surface model	Noah land surface model (Chen and Dudhia [48])
Microphysics scheme	Morrison double-moment scheme (Morrison et al. [49])
PBL scheme	Quasi-Normal Scale Elimination PBL scheme (Sukoriansky, et al. [50])
Horizontal resolution	27 km
Gas chemistry	MOZART (Emmons et al. [1]; Badia et al. [6])
Aerosol chemistry	MOSAIC (Zaveri et al. [2]; Badia et al. [6])
Photolysis	FTUV (Tie et al. [51]; Badia et al. [6])

Table S4 | WRF-Chem simulations design

Sources and reactions		Simulation scenarios						
		BASE	HAL	no_Cl_emi	no_Br_emi	no_$BrNO_2$_uptake	fixed_gamma	no_ClBr_SOA
Sources	Anthropogenic chlorine	-	Yes	-	Yes	Yes	Yes	Yes
	Anthropogenic bromine	-	Yes	Yes	-	Yes	Yes	Yes
	Oceanic halogen	-	Yes	Yes	Yes	Yes	Yes	Yes
Heterogeneous reactions of bromine species	$BrNO_2$ reactions:<br>$BrNO_2$ -> HOBr + HONO<br>$BrNO_2$ + HCl -> BrCl + HONO	-	$\gamma=0.001$<br>($rad < 0.01$) [a]<br>$\gamma=0.1$ ($0.01 < rad < 400$)<br>$\gamma=0.1*rad/400$<br>($rad > 400$)	$\gamma=0.001$<br>($rad < 0.01$)<br>$\gamma=0.1$ ($0.01 < rad < 400$)<br>$\gamma=0.1*rad/400$<br>($rad > 400$)	$\gamma=0.001$<br>($rad < 0.01$)<br>$\gamma=0.1$ ($0.01 < rad < 400$)<br>$\gamma=0.1*rad/400$<br>($rad > 400$)	-	$\gamma=0.1$	$\gamma=0.001$<br>($rad < 0.01$)<br>$\gamma=0.1$ ($0.01 < rad < 400$)<br>$\gamma=0.1*rad/400$<br>($rad > 400$)
	Other reactions:<br>HOBr + HCl -> BrCl + H_2O<br>HOBr + HBr -> BR_2 + H_2O<br>HOCl + HBr -> BrCl+H_2O<br>$ClNO_3$ + HBr ->BrCl + HNO_3<br>$BrNO_3$ + HCl -> BrCl + HNO_3<br>$BrNO_3$ ->HOBr+HNO_3	-	$\gamma=0.001$<br>($rad < 0.01$)<br>$\gamma=0.1$ ($0.01 < rad < 400$)<br>$\gamma=0.1*rad/400$<br>($rad > 400$)	$\gamma=0.001$<br>($rad < 0.01$)<br>$\gamma=0.1$ ($0.01 < rad < 400$)<br>$\gamma=0.1*rad/400$<br>($rad > 400$)	$\gamma=0.001$<br>($rad < 0.01$)<br>$\gamma=0.1$ ($0.01 < rad < 400$)<br>$\gamma=0.1*rad/400$<br>($rad > 400$)	$\gamma=0.001$<br>($rad < 0.01$)<br>$\gamma=0.1$<br>($0.01 < rad < 400$)<br>$\gamma=0.1*rad/400$<br>($rad > 400$)	$\gamma=0.1$	$\gamma=0.001$<br>($rad < 0.01$)<br>$\gamma=0.1$ ($0.01 < rad < 400$)<br>$\gamma=0.1*rad/400$<br>($rad > 400$)
Cl initiated SOA formation	Cl + VOC -> SOA	-	Yes	Yes	Yes	Yes	Yes	-
Br initiated SOA formation	Br + VOC -> SOA	-	Same SOA yield as Cl reaction	Same SOA yield as Cl reaction	Same SOA yield as Cl reaction	Same SOA yield as Cl reaction	Same SOA yield as Cl reaction	-
Other halogen reactions	Other Cl, Br, and I reactions	-	Yes	Yes	Yes	Yes	Yes	Yes

a: γ is the heterogeneous uptake coefficient. rad presents radiation in the unit of W/m^2.

Supplementary References:

1. Emmons, L. K., Walters, S., Hess, P. G., Lamarque, J.-F. F., Pfister, G. G., Fillmore, D., Granier, C., Guenther, A., Kinnison, D., Laepple, T., Orlando, J., Tie, X., Tyndall, G., Wiedinmyer, C., Baughcum, S. L. and Kloster, S.: Description and evaluation of the Model for Ozone and Related chemical Tracers, version 4 (MOZART-4), Geosci. Model Dev., 3(1), 43–67, doi:10.5194/gmd-3-43-2010, 2010.

2. Zaveri, R. A., Easter, R. C., Fast, J. D. and Peters, L. K.: Model for Simulating Aerosol Interactions and Chemistry (MOSAIC), J. Geophys. Res. Atmos., 113(13), 1–29, doi:10.1029/2007JD008782, 2008.

3. Shrivastava, M., Fast, J., Easter, R., Gustafson, W. I., Zaveri, R. A., Jimenez, J. L., Saide, P. and Hodzic, A.: Modeling organic aerosols in a megacity: Comparison of simple and complex representations of the volatility basis set approach, Atmos. Chem. Phys., 11(13), 6639–6662, doi:10.5194/acp-11-6639-2011, 2011.

4. Knote, C., Hodzic, A., Jimenez, J. L., Volkamer, R., Orlando, J. J., Baidar, S., Brioude, J., Fast, J., Gentner, D. R., Goldstein, A. H., Hayes, P. L., Knighton, W. B., Oetjen, H., Setyan, A., Stark, H., Thalman, R., Tyndall, G., Washenfelder, R., Waxman, E. and Zhang, Q.: Simulation of semi-explicit mechanisms of SOA formation from glyoxal in aerosol in a 3-D model, Atmos. Chem. Phys., 14(12), 6213–6239, doi:10.5194/acp-14-6213-2014, 2014.

5. Archer-Nicholls, S., Lowe, D., Utembe, S., Allan, J., Zaveri, R. A., Fast, J. D., Hodnebrog, Denier Van Der Gon, H. and McFiggans, G.: Gaseous chemistry and aerosol mechanism developments for version 3.5.1 of the online regional model, WRF-Chem, Geosci. Model Dev., 7(6), 2557–2579, doi:10.5194/gmd-7-2557-2014, 2014.

6. Badia, A., Reeves, C. E., Baker, A. R., Saiz-Lopez, A., Volkamer, R., Koenig, T. K., Apel, E. C., Hornbrook, R. S., Carpenter, L. J., Andrews, S. J., Sherwen, T. and von Glasow, R.: Importance of reactive halogens in the tropical marine atmosphere: a regional modelling study using WRF-Chem, Atmos. Chem. Phys., 19(5), 3161–3189, doi:10.5194/acp-19-3161-2019, 2019.

7. Odum, J.R., Hoffmann, T., Bowman, F., Collins, D., Flagan, R.C. and Seinfeld, J.H..: Gas/particle partitioning and secondary organic aerosol yields. Environ. Sci. Technol. 30, 2580-2585, 1996.

8. Murphy, B.N. and Pandis, S.N.: Simulating the formation of semivolatile primary and secondary organic aerosol in a regional chemical transport model. Environ. Sci. Technol. 43, 4722-4728, 2009.

9. Stanier, C. O., Donahue, N. and Pandis, S. N.: Parameterization of secondary organic aerosol mass fractions from smog chamber data, Atmos. Environ., 42(10), 2276–2299, doi:10.1016/j.atmosenv.2007.12.042, 2008.

10. Lane, T. E., Donahue, N. M. and Pandis, S. N.: Simulating secondary organic aerosol formation using the volatility basis-set approach in a chemical transport model, Atmos. Environ., 42(32), 7439–7451, doi:10.1016/j.atmosenv.2008.06.026, 2008.

11. Wang, D.S. and Hildebrandt Ruiz, L.: Secondary organic aerosol from chlorine-initiated oxidation of isoprene. Atmos. Chem. Phys. 17, 13491-13508, 2017.

12. Cai, X. and Griffin, R. J.: Secondary aerosol formation from the oxidation of biogenic hydrocarbons by chlorine atoms. J. Geophys. Res. Atmos. 111, 1–14, 2006.

13. Ofner, J., Kamilli, K. A., Held, A., Lendl, B. and Zetzsch, C.: Halogen-induced organic aerosol (XOA): A study on ultra-fine particle formation and time-resolved chemical characterization, Faraday Discuss., 165, 135–149, doi:10.1039/c3fd00093a, 2013.

14 Wang, D.S. and Hildebrandt Ruiz, L.: Chlorine-initiated oxidation of n-alkanes under high-NO x conditions: insights into secondary organic aerosol composition and volatility using a FIGAERO–CIMS. Atmos. Chem. Phys. 18, 15535-15553, 2018.

15. Dhulipala, S. V., Bhandari, S. and Hildebrandt Ruiz, L.: Formation of oxidized organic compounds from Cl-initiated oxidation of toluene, Atmos. Environ., 199 (November 2018), 265–273, doi:10.1016/j.atmosenv.2018.11.002, 2019.

16. Cai, X., Ziemba, L. D. and Griffin, R. J.: Secondary aerosol formation from the oxidation of toluene by chlorine atoms, Atmos. Environ., 42(32), 7348–7359, doi:10.1016/j.atmosenv.2008.07.014, 2008.

17. Peng, X., Wang, W., Xia, M., Chen, H., Ravishankara, A. R., Li, Q., Saiz-Lopez, A., Liu, P., Zhang, F., Zhang, C., Xue, L., Wang, X., George, C., Wang, J., Mu, Y., Chen, J. and Wang, T.: An unexpected large continental source of reactive bromine and chlorine with significant impact on wintertime air quality, Natl. Sci. Rev., doi:10.1093/nsr/nwaa304, 2020.

18. Fan, X., Cai, J., Yan, C., Zhao, J., Guo, Y., Li, C., Dällenbach, K. R., Zheng, F., Lin, Z., Chu, B., Wang, Y., Dada, L., Zha, Q., Du, W., Kontkanen, J., Kurtén, T., Iyer, S., Kujansuu, J. T., Petäjä, T., Worsnop, D. R., Kerminen, V.-M., Liu, Y., Bianchi, F., Tham, Y. J., Yao, L., and Kulmala, M.: Atmospheric gaseous hydrochloric and hydrobromic acid in urban Beijing, China: detection, source identification and potential atmospheric impacts, Atmos. Chem. Phys., 21, 11437–11452, https://doi.org/10.5194/acp-21-11437-2021, 2021.

19. Li, Q., Zhang, L., Wang, T., Tham, Y. J., Ahmadov, R., Xue, L., Zhang, Q. and Zheng, J.: Impacts of heterogeneous uptake of dinitrogen pentoxide and chlorine activation on ozone and reactive nitrogen partitioning: improvement and application of the WRF-Chem model in southern China, Atmos. Chem. Phys., 16(23), 14875–14890, doi:10.5194/acp-16-14875-2016, 2016.

20. Wang, X., Jacob, D., Fu, X., Wang, T., Le Breton, M., Hallquist, M., Liu, Z., McDuffie, E. and Liao, H.: Effects of anthropogenic chlorine on PM 2.5 and ozone air quality in China, Environ. Sci. Technol., doi:10.1021/acs.est.0c02296, 2020.

21. Hossaini, R., Chipperfield, M.P., Saiz-Lopez, A., Fernandez, R., Monks, S., Feng, W., Brauer, P. and von Glasow, R.: A global model of tropospheric chlorine chemistry: Organic versus inorganic sources and impact on methane oxidation. J. Geophys. Res. 121, 14271-14297, 2016.

22. Zhang, L., Li, Q., Wang, T., Ahmadov, R., Zhang, Q., Li, M. and Lv, M.: Combined impacts of nitrous acid and nitryl chloride on lower-tropospheric ozone: New module development in WRF-Chem and application to China. Atmos. Chem. Phys. 17, 9733–9750, 2017.

23. Liu, Y., Fan, Q., Chen, X., Zhao, J., Ling, Z., Hong, Y., Li, W., Chen, X., Wang, M. and Wei, X.: Modeling the impact of chlorine emissions from coal combustion and prescribed waste incineration on tropospheric ozone formation in China. Atmos. Chem. Phys. 18, 2709–2724, 2018.

24. Wang, X., Jacob, D.J., Eastham, S.D., Sulprizio, M.P., Zhu, L., Chen, Q., Alexander, B., Sherwen, T., Evans, M.J., Lee, B.H., Haskins, J.D., Lopez-Hilfiker F.D., Thornton J.A., Huey G.L., and Liao H.: The role of chlorine in global tropospheric chemistry. Atmos. Chem. Phys. 19, 3981–4003, 2019.

25. Li, Q., Badia, A., Wang, T., Sarwar, G., Fu, X., Zhang, L., Zhang, Q., Fung, J., Cuevas, C. A., Wang, S., Zhou, B. and Saiz-Lopez, A.: Potential Effect of Halogens on Atmospheric Oxidation and Air Quality in China, J. Geophys. Res. Atmos., 0–2, doi:10.1029/2019JD032058, 2020.

26. Fernandez, R. P., Salawitch, R. J., Kinnison, D. E., Lamarque, J.-F. F. and Saiz-Lopez, A.: Bromine partitioning in the tropical tropopause layer: Implications for stratospheric injection. Atmos. Chem. Phys. 14, 13391–13410, 2014.

27. Saiz-Lopez, A., Fernandez, R.P., Ordóñez, C., Kinnison, D.E., Gómez Martín, J.C., Lamarque, J.F. and Tilmes, S.: Iodine chemistry in the troposphere and its effect on ozone. Atmos. Chem. Phys. 14, 13119–13143, 2014.

28. Sarwar, G., Gantt, B., Schwede, D., Foley, K., Mathur, R. and Saiz-Lopez, A.: Impact of Enhanced Ozone Deposition and Halogen Chemistry on Tropospheric Ozone over the Northern Hemisphere. Environ. Sci. Technol. 49, 9203–9211, 2015.

29. Sherwen, T., Schmidt, J.A., Evans, M.J., Carpenter, L.J., Großmann, K., Eastham, S.D., Jacob, D.J., Dix, B., Koenig, T.K., Sinreich, R., Ortega, I., Volkamer R., Saiz-Lopez A., Prados-Roman C., Mahajan A.S., and Ordóñez C.: Global impacts of tropospheric halogens (Cl, Br, I) on oxidants and composition in GEOS-Chem. Atmos. Chem. Phys. 16, 12239–12271, doi:10.5194/acp-16-12239-2016, 2016.

30. Ianniello, A., Spataro, F., Esposito, G., Allegrini, I., Hu, M. and Zhu, T.: Chemical characteristics of inorganic ammonium salts in PM 2.5 in the atmosphere of Beijing (China). Atmos. Chem. Phys. 11, 10803-10822, 2011.

31. Chen, D., Liu, Z., Fast, J. and Ban, J.: Simulations of sulfate–nitrate–ammonium (SNA) aerosols during the extreme haze events over northern China in October 2014. Atmos. Chem. Phys., 16, 10707–10724, doi:10.5194/acp-16-10707-2016, 2016.

32. Chang, W.L., Brown, S.S., Stutz, J., Middlebrook, A.M., Bahreini, R., Wagner, N.L., Dubé, W.P., Pollack, I.B., Ryerson, T.B. and Riemer, N.: Evaluating N2O5 heterogeneous hydrolysis parameterizations for CalNex 2010. J. Geophys. Res. Atmos,. 121, 5051-5070, 2016.

33. Shah, V., Jaeglé, L., Thornton, J. A., Lopez-Hilfiker, F. D., Lee, B. H., Schroder, J. C., Campuzano-Jost, P., Jimenez, J. L., Guo, H., Sullivan, A. P., Weber, R. J., Green, J. R., Fiddler, M. N., Bililign, S., Campos, T. L., Stell, M., Weinheimer, A. J., Montzka, D. D. and Brown, S. S.: Chemical feedbacks weaken the wintertime response of particulate sulfate and nitrate to emissions reductions over the eastern United States, Proc. Natl. Acad. Sci., 115(32), 8110–8115, doi:10.1073/pnas.1803295115, 2018.

34. Wesely M. Parameterization of surface resistances to gaseous dry deposition in regional-scale numerical models. Atmos. Environ., 23, 1293–1304 (1989).

35. Liu, Z., Zhou, M., Chen, Y., Chen, D., Pan, Y., Song, T., Ji, D., Chen, Q. and Zhang, L.: The nonlinear response of fine particulate matter pollution to ammonia emission reductions in North China. Environ. Res. Lett. 16, 034014, 2021.

36. Nault, B. A., Jo, D. S., McDonald, B. C., Campuzano-Jost, P., Day, D. A., Hu, W., Schroder, J. C., Allan, J., Blake, D. R., Canagaratna, M. R., Coe, H., Coggon, M. M., DeCarlo, P. F., Diskin, G. S., Dunmore, R., Flocke, F., Fried, A., Gilman, J. B., Gkatzelis, G., Hamilton, J. F., Hanisco, T. F., Hayes, P. L., Henze, D. K., Hodzic, A., Hopkins, J., Hu, M., Huey, L. G., Jobson, B. T., Kuster, W. C., Lewis, A., Li, M., Liao, J., Nawaz, M. O., Pollack, I. B., Peischl, J., Rappenglück, B., Reeves, C. E., Richter, D., Roberts, J. M., Ryerson, T. B., Shao, M., Sommers, J. M., Walega, J., Warneke, C., Weibring, P., Wolfe, G. M., Young, D. E., Yuan, B., Zhang, Q., de Gouw, J. A., and Jimenez, J. L.: Secondary organic aerosols from anthropogenic volatile organic compounds contribute substantially to air pollution mortality, Atmos. Chem. Phys., 21, 11201–11224, https://doi.org/10.5194/acp-21-11201-2021, 2021.

37. Robinson, A.L., Donahue, N.M., Shrivastava, M.K., Weitkamp, E.A., Sage, A.M., Grieshop, A.P., Lane, T.E., Pierce, J.R. and Pandis, S.N.: Rethinking organic aerosols: Semivolatile emissions and photochemical aging. Science 315, 1259–1262, 2007.

38. Liu, J., Mauzerall, D.L., Chen, Q., Zhang, Q., Song, Y., Peng, W., Klimont, Z., Qiu, X., Zhang, S., Hu, M. and Lin, W., Smith K.R., and Zhu T.: Air pollutant emissions from Chinese households: A major and underappreciated ambient pollution source. Proc. Natl. Acad. Sci. 113, 7756-7761, 2016.

39. James, W. and Acevedo, L. Trace element partitioning in Texas lignite combustion. Journal of radioanalytical and nuclear chemistry, 171, 287-302, 1993.

40. Klein, D.H., Andren, A.W., Carter, J.A., Emery, J.F., Feldman, C., Fulkerson, W., Lyon, W.S., Ogle, J.C., Talmi, Y., van Hook R.I. and Bolton N.: Pathways of Thirty-seven Trace Elements Through Coal-Fired Power Plant. Environ. Sci. Technol. 9, 973–979, 1975.

41. Klika, Z., Bartoňová, L. and Spears, D. A. Effect of boiler output on trace element partitioning during coal combustion in two fluidized-bed power stations. Fuel 80, 907–917, 2001.

42. Peng, B. X., Li, L. and Wu, D. S. Distribution of bromine and iodine in thermal power plant. Journal of Coal Science and Engineering 19, 387–391, 2013.

43. Song, D., Qin, Y., Zhang, J., Wang, W. and Zheng, C.: Distribution of environmentally-sensitive trace elements of coal in combustion. Coal Conversion, 28, 56-60, 2005.

44. Wang, W. F., Qin, Y. and Song, D. Y. Study on the mobility and release of trace elements in coal-fired power plant. Acta Sci. Circumstantiae 23, 748–752, 2003.

45. Lei, Y., Zhang, Q., Nielsen, C. and He, K. An inventory of primary air pollutants and CO2 emissions from cement production in China, 1990-2020. Atmos. Environ. 45, 147–154, 2011.

46. Peng, B. and Wu, D. Study on bromine release from bituminous coal during combustion. Fuel 157, 82–86, 2015.

47. Iacono, M.J., Delamere, J.S., Mlawer, E.J., Shephard, M.W., Clough, S.A. and Collins, W.D.: Radiative forcing by long-lived greenhouse gases: Calculations with the AER radiative transfer models. J. Geophys. Res. Atmos. 113, 2–9, 2008.

48. Chen, F. and Dudhia, J. Coupling an Advanced Land Surface–Hydrology Model with the Penn State–NCAR MM5 Modeling System. Part II: Preliminary Model Validation. Mon. Weather Rev. 129, 587–604, 2001.

49. Morrison, H., Thompson, G. and Tatarskii, V. Impact of Cloud Microphysics on the Development of Trailing Stratiform Precipitation in a Simulated Squall Line: Comparison of One- and Two-Moment Schemes. Mon. Weather Rev. 137, 991–1007, 2008.

50. Sukoriansky, S., Galperin, B. and Perov, V. Application of a new spectral theory of stably stratified turbulence to the atmospheric boundary layer over sea ice. Boundary-Layer Meteorol. 117, 231–257, 2005.

51. Tie, X. Effect of clouds on photolysis and oxidants in the troposphere. J. Geophys. Res. 108, 4642, doi:10.1029/2003JD003659, 2003.
